# ProtFus: A Comprehensive Method Characterizing Protein-Protein Interactions of Fusion Proteins

**DOI:** 10.1371/journal.pcbi.1007239

**Published:** 2019-08-22

**Authors:** Somnath Tagore, Alessandro Gorohovski, Lars Juhl Jensen, Milana Frenkel-Morgenstern

**Affiliations:** 1 The Cancer Genomics and BioComputing of Complex Diseases lab, Azrieli Faculty of Medicine, Bar-Ilan University, Safed, Israel; 2 Cellular Network Biology Group, Novo Nordisk Foundation Center for Protein Research, Faculty of Health and Medical Sciences, University of Copenhagen, Copenhagen, Denmark; Fox Chase Cancer Center, UNITED STATES

## Abstract

Tailored therapy aims to cure cancer patients effectively and safely, based on the complex interactions between patients' genomic features, disease pathology and drug metabolism. Thus, the continual increase in scientific literature drives the need for efficient methods of data mining to improve the extraction of useful information from texts based on patients' genomic features. An important application of text mining to tailored therapy in cancer encompasses the use of mutations and cancer fusion genes as moieties that change patients' cellular networks to develop cancer, and also affect drug metabolism. Fusion proteins, which are derived from the slippage of two parental genes, are produced in cancer by chromosomal aberrations and trans-splicing. Given that the two parental proteins for predicted fusion proteins are known, we used our previously developed method for identifying chimeric protein–protein interactions (ChiPPIs) associated with the fusion proteins. Here, we present a validation approach that receives fusion proteins of interest, predicts their cellular network alterations by ChiPPI and validates them by our new method, ProtFus, using an online literature search. This process resulted in a set of 358 fusion proteins and their corresponding protein interactions, as a training set for a Naïve Bayes classifier, to identify predicted fusion proteins that have reliable evidence in the literature and that were confirmed experimentally. Next, for a test group of 1817 fusion proteins, we were able to identify from the literature 2908 PPIs in total, across 18 cancer types. The described method, ProtFus, can be used for screening the literature to identify unique cases of fusion proteins and their PPIs, as means of studying alterations of protein networks in cancers.

Availability: http://protfus.md.biu.ac.il/

## Author summary

Tailored therapy aims to cure cancer patients in a fully personal way. Thus, the continual increase in scientific information and, particularly, in published literature, drives the need for efficient methods of data mining to find unique personal genomic features and their connections. Fusion proteins, which are derived from the slippage of two parental genes or chromosomal translocations, are frequently drivers of cancers. We used our previously developed method for identifying chimeric protein–protein interactions (ChiPPIs) for multiple fusion proteins. In this paper, we present a validation approach, ProtFus, which receives fusion proteins of interest, predicts their cellular network alterations by ChiPPI and validates them by online literature searches. This process resulted in a set of 358 fusion proteins and their corresponding protein interactions, as training set. Next, for a test set of 1817 fusion proteins, we were able to identify 2908 previously published PPIs across 18 different cancer types. The described method can be used for screening the literature to identify unique cases of fusion proteins and their PPI networks, as means of studying alterations of protein networks for personalized approaches in cancers.

This is a *PLOS Computational Biology* Methods paper.

## Introduction

### Background

Fusion proteins resulting from chromosomal translocations have important roles in several types of cancer and are extensively discussed in cancer research literature. The current biomedical literature resources, such as PubMed, comprise more than 28 million citations, with approximately 14,000 cancer-related papers from 2018 alone, and more than 3 million abstracts in total that mention ‘cancer’. Similarly, the number of PubMed articles that mention ‘fusion proteins’ is also increasing rapidly. Thus, there is a growing need to catalog as well as curate this information. Hence, text mining-based methods to identify fusion proteins from PubMed are extremely important. Moreover, information regarding fusion proteins mentioned in the current literature have no standard format. The upshot is that identifying a certain fusion protein is non-trivial; for example, a fusion protein such as BCR–ABL1 is represented in variable forms in different texts [1–2]. These variations include the formatting of the fusion events themselves (e.g., BCR-ABL1 vs. BCR:ABL1 vs. BCR/ABL1), and the keywords used to describe them (fusions vs. fusion proteins vs. chimeric proteins vs. chimeras) [3]. Moreover, when extracting protein–protein interactions (PPIs) of fusions, their actions can be described in varying ways (e.g., activate vs. interact vs. express vs. induce [4]).

To collect information about multiple fusion proteins, we developed an in-house database, ChiTaRS [1], which covers more than 11,000 cancer breakpoints. We continually mine the new literature for mentions of fusion proteins, their parent proteins and their associated PPI networks, so as to provide a constantly updated fusion protein database tool for the scientific community worldwide.

### Previous studies in the field

Text mining is used in biology to reveal associations between genes and proteins, as described in the literature. Several earlier studies focused on developing text-mining approaches for modern medical text, in general, and cancer research, specifically. For example, several annotated corpora have been created to distinguish mutations, cancer processes, tumor suppressors, oncogenes and transcription factors [5–7]. Natural language processing (NLP) methods use named-entities that have different sequences/phrases of nouns and adjectives, while named-entities involved in relationships are designated by verbs. Syntactic analysis may be defined as the process of analyzing by NLP the strings of symbols that conform to the rules of formal language grammar. Further, several previous and currently available tools enable extracting a specific set of information from the literature. Examples of such biomedical text mining tools are: MetaMap [8], WhatIzIt [9], iHOP [10], PubTator [11] and Gimli [12]. Moreover, to provide a constantly up-dated resource for research purposes, continuous evaluations and verifications are performed by the biomedical text mining community through multiple data assessment initiatives, e.g. BioCreative [13–14], BioNLP [15] and i2b2 [16], to name a few. Therefore, NLP and text mining of medical literature is a growing field that may provide a novel resource in fusion moieties and their cellular processes.

Screening for fusion proteins and their PPIs is a relatively new field in biomedical text mining, and as such, only a limited number of relevant resources are available [17–18]. Some well-known databases of fusion proteins have been developed, such as ChiTaRS-3.1 [1], ChimerDB 3.0 [19], COSMIC [20] and TICdb [21]. However, no available biomedical resource can automatically extract the PPIs of fusion proteins that have been confirmed experimentally from scientific papers. Moreover, given that the two parental proteins for predicted fusion proteins were known, our previously developed method may be used to predict chimeric PPIs (ChiPPIs) associated with the fusion proteins [22]. Here, we present a text-mining approach, called Protein Fusions Server (ProtFus) that receives fusion proteins of interest, predicts their cellular network alterations by ChiPPI [22] and validates them by searching PubMed references. ProtFus mines the literature to identify unique cancer fusion proteins and their experimentally described PPIs in scientific publications. Thus, ProtFus provides unique ways for extracting and interpreting information present in public scientific resources. Our objectives and process for employing ProtFus were as follows:

1. We aimed to identify fusion proteins and their interactions, from published scientific articles [23–24]. From the point-of-view of text mining, this task deals with identifying information that must be tagged to find co-mentions, like "human fusion proteins" with "cancer" or "cancers" and with "interactions" or "interactors" *etc*. Assuming that we are interested in the fusion protein BCR-ABL1, we will want to find all the mentions of BCR-ABL1 in the literature. But, BCR-ABL1 can be written in different forms (see above); thus, we looked for a specific ‘tagger’ that can identify all the possible forms.
2. Identifying interaction co-occurrences with fusion proteins is more complicated since it requires tagging “interaction tokens” from the literature and linking them to their correct fusion proteins. An example is the text ‘Grb2 has been shown to bind NPM-ALK and ATIC-ALK in previous works using the interaction token that was ‘*bind*’.’
3. We developed ProtFus, a new online server, which identified instances of fusion proteins and their interactions from the literature [25], based on text mining approaches using NLP methods [26]. The major goal was to identify the co-occurrences of both fusion proteins and their corresponding interactions by filtering out the false positive cases from general searches using PubMed, such that a more focused result may be generated.

Thus, ProtFus can be used to validate from the biomedical literature, protein interactions of fusion proteins in cancer, which can then be empirically tested. Furthermore, the interactions can be used to validate the predicted ChiPPI networks [22] of multiple fusion proteins in different public databases.

## Methods

**Fig 1:**
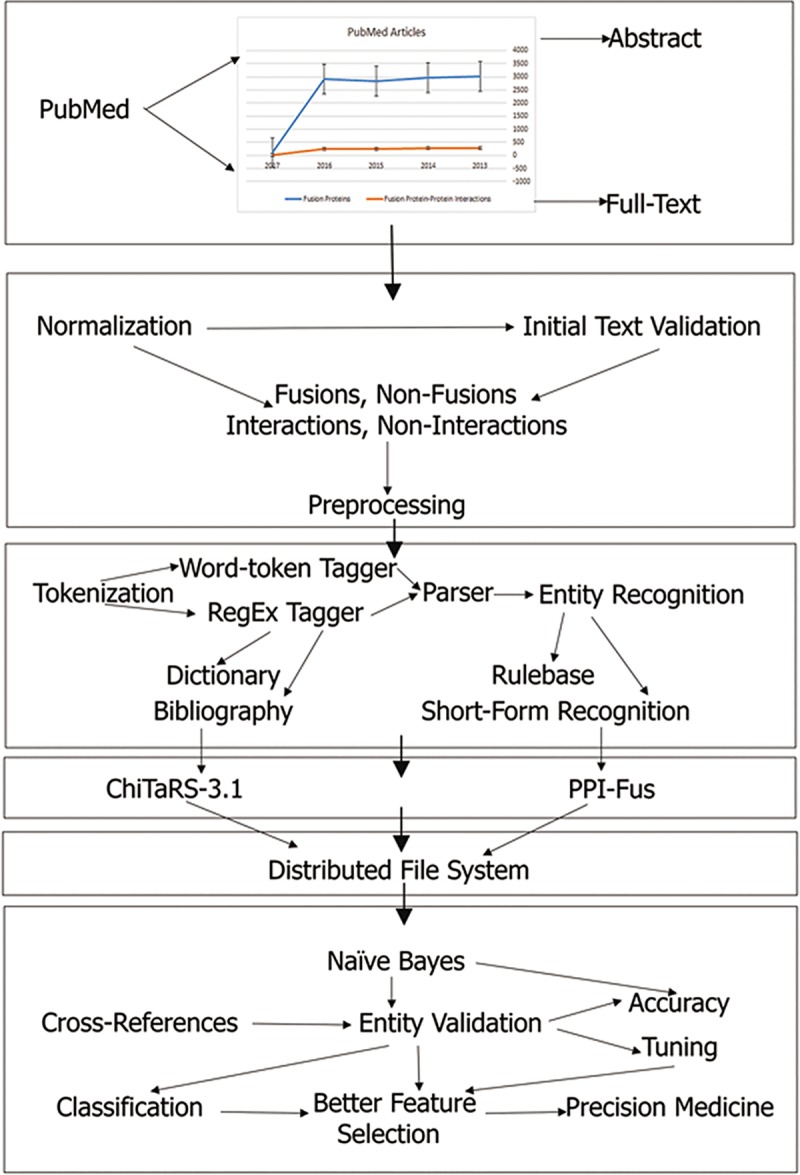
The overall methodology of ProtFus. The algorithm begins with collecting abstracts and full-texts from PubMed, followed by normalization, tokenization and entity recognition, cross-references, databases, and machine learning classifier.

The basic framework of ProtFus is depicted in (***Fig 1***). It consists of basic computational methods, such as text mining, machine learning and a distributed database system for storing the text, as well as features extracted from biomedical literature. Here, we explain the development of ProtFus to extract fusion protein information (e.g., BCR-ABL1), cancer type (e.g., BCR-ABL1 that causes chronic myelogenous leukemia) and prediction models (e.g., Naïve Bayes) for classifying text extracted from PubMed references. We also used a list of the cancer fusion proteins from the Mitelman Database of Chromosome Aberrations and Gene Fusions in Cancer [27], and the Cancer Breakpoints Collection of the ChiTaRS-3.1 database [1] (**Tables 1 and 2**) to validate fusion proteins obtained by text mining of biomedical literature.

**Table 1:** Datasets considered for training. (collected from PubMed between January 2013 and April 2017).

PubMed Year	Abstracts	Full Texts	“Fusion proteins”	“Fusion proteins”+PPI
2017	17220	5212	43	2
2016	352097	163884	1164	99
2015	353972	171432	1132	104
2014	321314	156091	1187	112
2013	299380	141512	1203	110

**Table 2:** Datasets considered for testing ProtFus.

PubMed Year	Abstracts	Full Texts	”Fusion proteins”	“Fusion proteins”+PPI
2017	25830	7819	65	5
2016	528146	245826	1747	148
2015	530960	257148	1697	155
2014	481971	234136	1780	167
2013	449069	212268	1805	165

### Initial text validation

The initial text validation was performed on input from PubMed to remove false positive results, followed by segregation into tokens. We performed stemming of words for sentences, followed by identifying named-entities within sentences with the ‘Porter2’ algorithm, using the ‘stemming’ package in Python [28]. The named-entities within sentences were blanked out to make them more generalized. This step was followed by using a bag-of-words representation [29] based on a frequency score (*FS*) for estimating the importance of selecting a token. For the bag-of-words representation, we used the *FS* threshold (*T*_*s*,*a*_) (Eq 1):

$$T_{s,a}=FS_{s,a}\times \log_{10}\left(\frac{\tau }{\sigma }\right)$$ (Eq 1)

Here, *FS*_*s*,*a*_ denotes the frequency of token *s* in article *a*, *τ* is the number of articles/abstracts, *σ* is the number of articles having *s*. This threshold is used to estimate the frequency score. We used the Naïve Bayes classification method to build the PPI extraction model [29]. This categorizes the tokens in abstracts and articles to either fusion proteins or interactions of fusion proteins, and assigns them to Medical Subject Headings (MeSH) terms. The ProtFus framework was developed on an Apache Dell R820 server, with 1TB RAM and with a back-end My-SQL database and 1PB of support data from ChiTaRS-3.1 [1]. The tool was developed using Python, whereas the interface was developed using CGI-Perl (http://protfus.md.biu.ac.il/).

### Feature extraction

**Fig 2:**
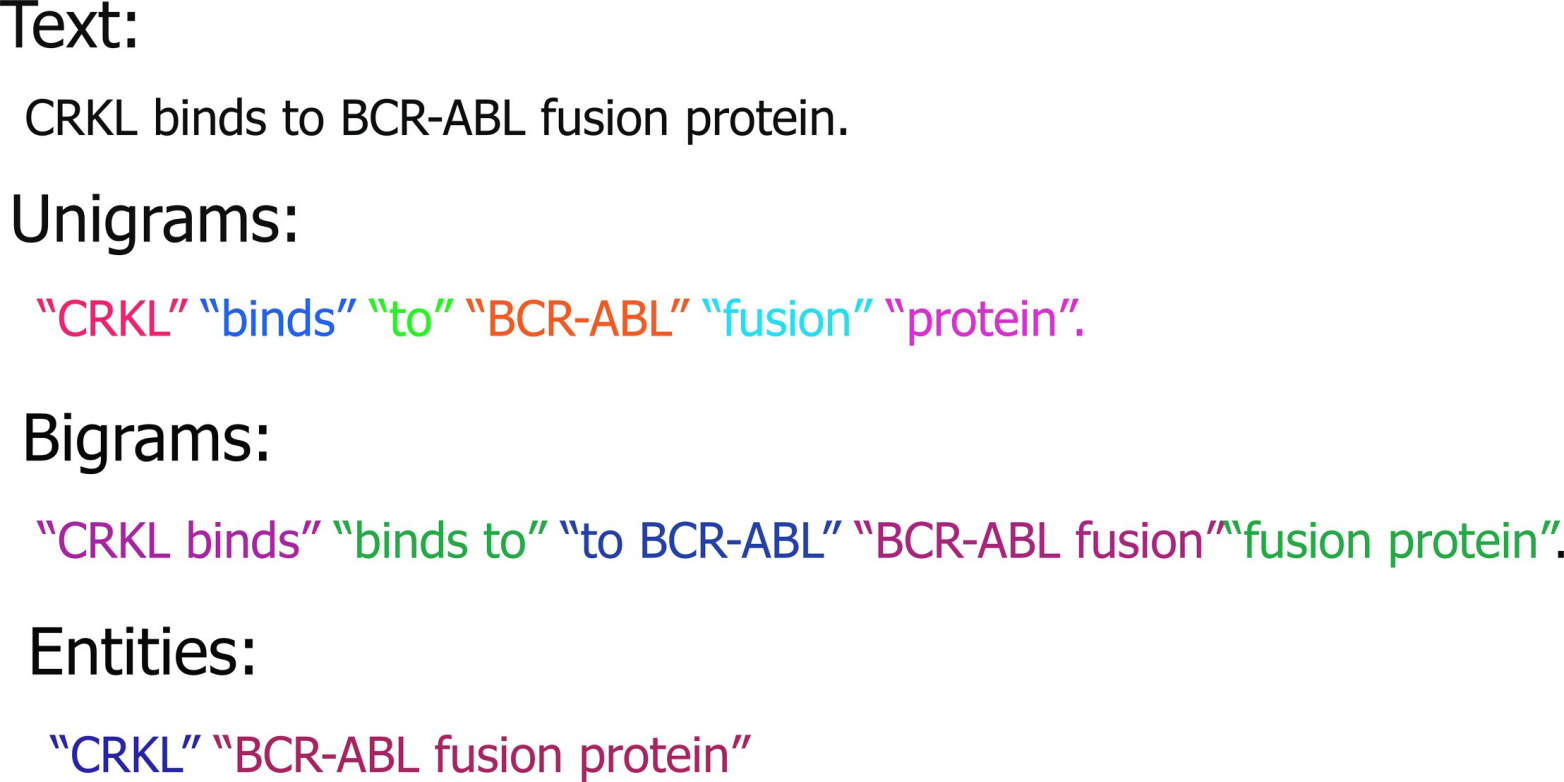
N-gram model for detecting N-words by ProtFus. The N-gram model and some possible sets of combinations.

We used the N-gram model for detecting N-words at a time from a given sentence. An N-gram model is a model of "strings" or "sequences" in NLP by means of the statistical properties of N-grams, based on the appearance of letters, according to the Shannon information theory of likelihood [30]. Specifically, using a 2-gram method, all words in a sentence were broken down into two combinations, including unigrams and bigrams, i.e., one- and two-word combinations [31]. For example, some possible sets of combinations were provided in Fig 2. We extracted a set of bigrams, as well as combinations of 3- and 4-grams, from abstracts or full-text articles in order to train ProtFus to detect specific fusion protein instances. In addition, the instances of these tokens were counted in the back-end corpus [32]. A back-end text corpus was a structured set of texts that can be used for statistical analysis; it checks occurrences and validates linguistic rules in a specific context. In our case, the back-end corpus was used for performing background feature extraction using N-grams. Further, when *FS* was the standard feature score, a considerably high threshold (*T*_*s*,*a*_) was given to tokens that appeared frequently in the corpus. Moreover, we also converted all abstracts or full-text articles into ‘similar-length’ feature vectors, where each feature represents *T*_*s*,*a*_ of the identified token. The rationale was that these feature vectors are further used for rescaling the overall feature score.

Subsequently, we organized a bag-of-words representation of the feature vectors (***Table 3***). A bag-of-words was a representation of text that described the occurrence of words within a document. Its two components are a vocabulary of known words and a measure of the presence of known words. Thus, ***S1–S2 Tables (Supporting information)*** include the back-end corpus considered for tagging fusion proteins and their interactions. The word-token tagger had a back-end Synonyms (with synonyms resource, ***S3 Table*, *Supporting information***), whereas the RegEx tagger had a back-end Synonyms (with rulebase, ***S4 Table*, *Supporting information***). Likewise, ***Table 4*** represents Precision and Recall for a retrieval step.

**Table 3:** Bag-of-words collection for 10 PubMed ID abstracts.

PMID	Fusion proteins	Fusion Gene	Biological Token	Miscellaneous Token
24186139	1	1	20	35
22101766	0	1	25	30
18451133	0	1	28	38
11930009	1	1	26	32
15735689	0	1	21	34
18850010	0	0	27	33
21193423	1	0	23	33
22570737	1	0	30	38
18383210	1	0	29	35
24345920	1	0	26	32
16502585	1	0	21	33

**Table 4:** Precision and Recall for retrieval step.

Dataset	Precision	Recall	F-Score	Accuracy
Set A	0.79	0.82	0.76	0.81
Set B	0.81	0.83	0.78	0.80
Set C	0.85	0.84	0.82	0.85
Set D	0.72	0.76	0.72	0.74
Set E	0.80	0.82	0.78	0.82
Set F	0.81	0.81	0.78	0.82
Set G	0.78	0.83	0.81	0.83
Set H	0.75	0.81	0.78	0.80
Set I	0.85	0.82	0.81	0.83
Set J	0.73	0.78	0.76	0.75

### Named-entity recognition

The tokens were used to parse the texts for performing named-entity recognition (NER) [33]. NER locates and classifies named-entities in text into pre-defined categories. For example, the unannotated block of text ‘CRKL binds to BCR-ABL fusion protein’ can be annotated as ‘[CRKL] _protein_ binds to [BCR] _protein_—[ABL] _protein_ [fusion protein] _key_’. This was followed by searching for a pattern like protein1-protein2 key or protein1/protein2 key or protein1:protein2 key (e.g., [BCR] _protein_—[ABL] _protein_ [fusion protein] _key_). To associate a fusion event with a certain cancer we performed NER of ‘diseases’. For example, in ‘BCR-ABL causes leukemia’, we performed annotations such as ‘[BCR] _protein_-[ABL] _protein_ [causes] _action-verb_ [leukemia] _cancer_’. The ProtFus method performs a search in PubMed abstracts or uploads a full text file that is based on a specific input text. For example, in the case of an input text, the result is displayed in a separate pop-up window, and the fusion proteins are highlighted. Similarly, in the case of PPIs among fusion proteins, the result window includes the input text, and the interactions are highlighted. Thus, another feature of ProtFus is direct searching using PubMed articles. Users can select from the drop-down menu of 100, 200 or more, the number of articles to be considered for searching fusion proteins and their interactions. The result includes the abstracts of the articles that match best with fusion protein keywords (e.g., for BCR-ABL). This file can be further used for highlighting the fusion proteins and their interactions. Thus, ***Table 5*** represents Precision and Recall for NER.

**Table 5:** Precision and Recall for named-entity recognition.

Dataset	Precision	Recall	F-Score	Accuracy
Set A	0.79	0.82	0.77	0.81
Set B	0.77	0.82	0.80	0.82
Set C	0.87	0.83	0.82	0.89
Set D	0.80	0.81	0.76	0.78
Set E	0.84	0.83	0.82	0.82
Set F	0.81	0.84	0.83	0.83
Set G	0.81	0.89	0.85	0.84
Set H	0.82	0.82	0.84	0.80
Set I	0.82	0.84	0.83	0.87
Set J	0.78	0.80	0.77	0.79

### Designing a model for training and testing

We downloaded abstracts from PubMed to generate both training and test datasets. For a training set we used several datasets (***Table 1***). Other datasets served as test sets to evaluate the model that was built and a 10-fold cross-validation was performed, each time using 40% of the entities to train an extraction model and the remaining 60% to test it [33] (see ***Supporting information***).

### The NLP tokenization of bag-of-words

Tokenization is the task of chopping a character sequence and a defined document unit into pieces, called tokens, while perhaps throwing away certain characters, such as punctuation. Tokenization was performed using two specific taggers:

1. Word-token tagger
2. RegEx tagger

The word-token tagger identified the property of words from the text for fusion proteins, like ‘fusion proteins’, ‘fusion transcripts’, ‘chimeric proteins’, ‘chimeric genes’ and ‘fusion gene transcripts’; and "*action words*" for PPIs, like ‘activate’, ‘block’, ‘depend’, ‘express’ and ‘interact’. Similarly, the RegEx tagger recognizes and associates these word-tokens with their corresponding “literals” (attributes). The tokenizer module segregates the text into ‘Biological’, ‘Miscellaneous’, ‘Function’ and ‘Literal’ tokens. For example, given the following text, “The small molecule BCR-ABL-selective kinase inhibitor imatinib is the single most effective medical therapy for the treatment of chronic myeloid leukemia”, the tokenization output is: Biological Tokens—‘small’, ‘BCR-ABL-selective’, ‘single’, ‘medical’ and ‘chronic’; Miscellaneous Tokens—‘molecule’, ‘kinase’, ‘imatinib’, ‘therapy’, ‘treatment’, ‘myeloid’ and ‘leukemia’; Function Tokens—‘effective’ and ‘inhibitor’; Literal Tokens—‘is’, ‘the’, ‘for’ and ‘of’.

### Entity recognition from fusion and PPI corpus

Here, we present the structure of the corpus that was used for validation and testing. ProtFus considered all possible combinations of representing fusion proteins in text, by looking the back-end Rule-base as well as the fusion and PPI corpus. Now, we define the different keywords and tokens used by our method, as part of entity recognition. The back-end ‘*Synonyms’* (*fusion corpus*) consists of ‘entity’ ‘relation token’, such as ‘fusion’ ‘fusions, fusion transcript, fusion transcripts, fusion protein, fusion proteins, fusion gene, fusion genes’, whereas ‘*Synonyms’* (*PPI corpus*) consists of ‘entity’ ‘relation token’, such as ‘Activate’ ‘activate, activates, activated, activating, activation, activator’. Similarly, the back-end ‘*Synonyms’* (*fusion*) consists of ‘Fusion proteins’ ‘Synonyms’ ‘Alternate representations’, such as ‘EWS-FLI1’ ‘TMRPSS2-ERG’ ‘ews: fli1, EWSR1: EWS, EWSR1/FLI1, EWS/FLI-1’. The ‘*parser’* and the entity recognition module used ‘Rule-base’ and ‘Short Form Recognition’ back-end resources for identifying the final ‘best-suited’ entities and tokens, and also for filtering out the false positives. The ‘*Rule-base*’ (for *normalization*) consisted of ‘Rule’ ‘Input’ ‘Output’ ‘Reg Ex’, such as ‘Normalization of case’ ‘BCR-ABL, bcr-abl, BCR:ABL, bcr:ABL, BCR/ABL, bcr/abl’. Similarly, the ‘*Rule-base*’ (for *regular expression*) consisted of ‘Characteristics’ ‘Description’ ‘Rule’ ‘Reg Ex’, such as ‘Fusion token’ ‘Tokens with fusion word occurrence’ should be separated by space/tokens (‘fusion|fusions|fusion genes|gene fusion|fusion protein|fusion transcripts’) etc.

## Results

The fusion protein information extracted by ProtFus was validated using the ChiTaRS-3.1 [1] database of known cases of fusion proteins and interactors, as well as the Mitelman database of Chromosome Aberrations and Gene Fusions in Cancer [27]. The fusion protein occurrences, as predicted by ProtFus, were validated by searching the corresponding occurrences of breakpoints in cancers from the ChiTaRS database [1]. The Mitelman database was used mainly for identifying potential fusion proteins and their roles in cancer. Finally, the PPIs predicted by ChiPPI [22] were validated by ProtFus. The interactions of information that we received were compared with that of ChiPPI [22] and STRING [34–35], by performing simultaneous searches in both of these for cross-validation of the reliability of the results. We were particularly interested in searching for instances of interactions from the scientific literature.

Next, ProtFus was tested on 358 fusion proteins (based on the Mitelman Database) from PubMed articles; and the result statistics of the top 100 fusion proteins were provided based on their identification from the text (***S5 Table*, *Supporting information***). For example, in the case of the BCR-ABL1 fusion protein (PubMed ID = 9747873), ProtFus identified its occurrence in all PubMed articles, like, ‘Both Bcr-Abl fusion proteins exhibit an increased tyrosine kinase activity and their oncogenic potential has been demonstrated using in vitro cell culture systems as well as in in vivo mouse models’ (***S5 Table*, *Supporting information***). Similarly, ProtFus also identified interactions among fusion proteins (***S6 Table*, *Supporting information***), such as in the case of the BCR-ABL1 fusion protein (PubMed ID = 9747873), ‘The SH2-containing adapter protein GRB10 interacts with BCR-ABL’ (***S6 Table*, *Supporting information***). Particularly, the essential parameters for examining the accuracy of text-mining based algorithms involved the identification of Precision, Recall and F-Score. Moreover, ProtFus identified fusion proteins with Precision ranging from 0.33 to 1.0 (average = 0.83), Recall = 0.4 to 1.0 (average = 0.84) and F-Score = 0.4 to 1.0 (average = 0.81); whereas for PPIs with: Precision = 0.42 to 1.0 (average = 0.81), Recall = 0.5 to 1.0 (average = 0.81) and F-Score = 0.59 to 1.0 (average = 0.83). A high scoring system would typically have a Precision of ~0.8–1, Recall of ~0.8–1 and F-score of ~0.8–1, depending on the quality of data. Thus, the overall accuracy of ProtFus enabled extracting different attributes of fusion proteins and their interaction appearances in the biomedical texts.

### Training and testing

We used a classical Naïve Bayes algorithm for training as well as extraction. The datasets were partitioned based on known fusion proteins and their interactors from the literature. This resulted in a training set (40%) (***Table 1***) and a set (around 60%, when there was no reported fusion) that was used for testing the algorithm in all PubMed references (2013–2017) (***Table 2***). There was no overlap between training and testing data. Subsequently, decisions were modeled for assigning labels to raw input data. This type of classification algorithm can also be thought of as a convex optimization problem, in which one needs to identify the minima of a convex function *ρ*, associated with an input vector *v*, having *n* entries (Eq 2),

$$\min\left(\rho (v)\right)v\in Z^{n}$$ (Eq 2)

Here, the objective function can be defined as Eq 3,

$$\rho (v)=Z^{n}+\frac{1}{n}\sigma_{i=1}^{n}\mu \left(v;a(i),b(i)\right)$$ (Eq 3)

where vectors *a*(*i*)∈*Z^n^* are training instances (1≪*n*),*y*(*i*)∈*Z^n^* that act as labels. To examine the accuracy of our algorithm, we performed a 10-fold cross-validation. For this purpose, we partitioned the input text into ten equal-sized sub-samples, of which five were retained for testing and five were used for model building. We also used the standard Precision, Recall and F-score values for validating the results. Precision *(P)* was defined as the fraction of retrieved instances that was relevant to the study. Precision can also be defined as the probability that randomly selected retrieved information is relevant (Eq 4).

$$P=\frac{TP}{TP+FP}$$ (Eq 4)

Here, *TP* = true positive and *FP* = false positive. Similarly, Recall (*R*) is defined as the fraction of relevant instances that are retrieved for the study. Recall can also be defined as the fraction of the information relevant to the query that is successfully retrieved (Eq 5).

$$R=\frac{TP}{TP+FN}$$ (Eq 5)

Here, *FN* = false negative. Finally, *F-score* is the harmonic means of precision and recall (Eq (6).

$$F-score=2\left[\frac{P\cdot R}{P+R}\right]$$ (Eq 6)

For example, if the standard query text contains 3 tokens that could be categorized as fusion proteins, and ProtFus identifies 2 of them, the accuracy can be calculated as: True (standard) tokens = *n*, *y*, *n*, *a*; Predicted (by ProtFus) tokens = *n*, *n*, *n*, *a* (here, *n* = no token instance, *y* = token instance, *a* = noise). In this case, *Precision* = 0.75, *Recall* = 0.75 and *F-score* = 0.75. Similarly, the corresponding accuracy plot can be drawn by providing information about *Precision*, *Recall*, and *F-score* values, and the number of runs. ProtFus still had a high false-positive rate, due to the diverse corpus of texts and different forms of fusion mentions. However, this rate automatically decreased when the corpus was updated with better literals.

### Big Data processing using ProtFus and ChiPPI

To display the results of ProtFus in a user-friendly manner, we also built the Protein-Protein Interaction of Fusions (PPI-Fus) database (http://protfus.md.biu.ac.il/bin/protfusdb.pl), supported by Apache Tomcat and My-SQL. This is an open source Big Data processing framework that supports ETL (Extract, Transform and Load) and machine learning, as well as graph generation. Some classical text mining tasks can also be performed by identifying biological, functional, literal and miscellaneous tokens, as well as chunks from text. Further, for the purpose of entity recognition, the word-token tagger has back-end Synonyms (with a synonym resource), whereas the RegEx tagger has back-end synonyms (with rule base).

Further, in the case of identifying PPIs among fusion proteins, the pop-up result window included the input text with interactions highlighted. Another feature of ProtFus is direct searching using PubMed articles. Users can select from the given drop-down box, the number of articles to be considered for searching fusion proteins and their interactions. The result includes the abstracts of all the articles that best match with fusion protein keywords. This file can be further used for highlighting the fusion proteins and their interactions.

Interestingly, the *biological* tokens correspond mainly to nouns; *miscellaneous* tokens may correspond to verbs, pro-verbs, adverbs. *etc*; *function* tokens correspond to verbs and adjectives; and *literals* correspond to conjunctions. ***Tables 4*** and ***5*** represent the Precision and Recall for the retrieval step and NER, respectively (see Methods). Similarly, ***Table 6*** provides the overall accuracy of the Naïve Bayes classifier, whereas ***Table 7*** represents a comparative analysis of the overall extraction rate of fusion proteins and their PPIs using ProtFus and a selection of other resources. This comparison showed that ProtFus performs much better in overall extraction, with 92% accuracy. Thus, the process of tokenization was a very important step in our script, as it filtered out essential tokens (like protein and function tokens) from non-essential ones (like miscellaneous and literals) for better data extraction.

**Table 6:** Accuracy score of classifiers.

Dataset	Precision	Recall	F-Score	Accuracy
Set A	0.82	0.86	0.79	0.84
Set B	0.83	0.85	0.82	0.83
Set C	0.91	0.92	0.89	0.91
Set D	0.79	0.81	0.74	0.77
Set E	0.86	0.85	0.83	0.85
Set F	0.85	0.85	0.83	0.84
Set G	0.85	0.87	0.84	0.85
Set H	0.81	0.83	0.83	0.82
Set I	0.87	0.86	0.84	0.86
Set J	0.75	0.81	0.79	0.78

**Table 7:** Performance of ProtFus compared to other resources.

Resource	Full-Text	Extraction
ChimerDB-3.0	Yes	82%
FusionCancer (does not use text mining)	Yes	NA
FusionDB (does not use text mining)	Yes	NA
ProtFus	Yes	92%

**Fig 3:**
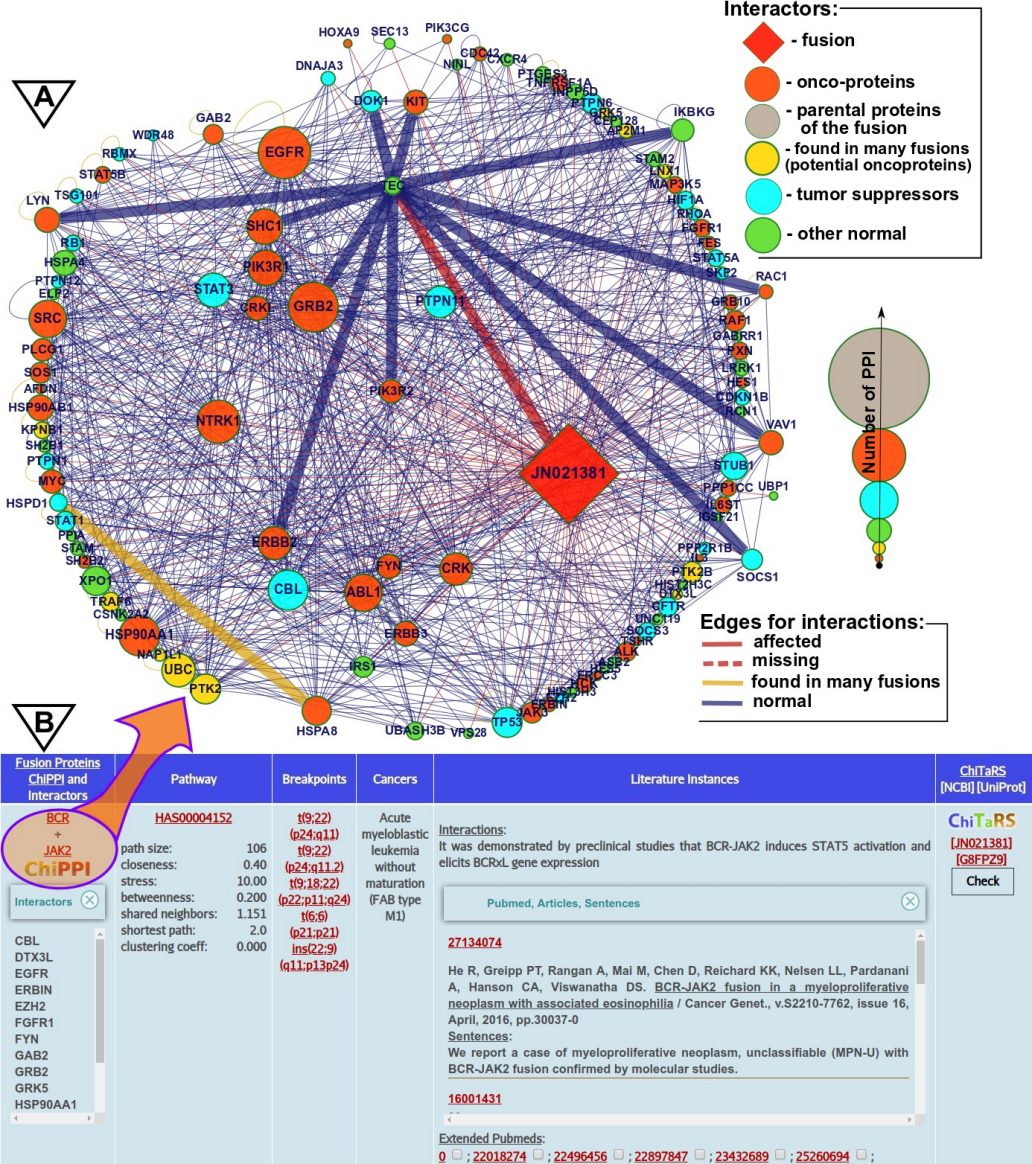
ChiPPI analysis (a) PPI-Fus/ProtFus extraction for BCR-JAK2 and STAT5B interaction (b) as predicted by ProtFus.

Considering discrete protein domains as binding sites for specific domains of interacting proteins, we catalogued the protein interaction networks of more than 11,000 cancer fusions in order to predict PPIs of fusion proteins using ChiPPI [22]. Mapping the effects of fusion proteins on cell metabolism and protein interaction networks reveals that chimeric PPI networks often lose tumor suppressor proteins and gain onco-proteins. As a case study, we compared the results generated by ProtFus with the interaction prediction accuracy of ChiPPI [22]. For example, in BCR-JAK2 fusion, ProtFus provided multiple hits regarding its occurrence in literature, such as, “It was demonstrated in preclinical studies that BCR-JAK2 induces STAT5 activation that elicits BCRxL gene expression” (PMC3728137), as correctly predicted by ChiPPI (***Fig 3***).

### ProtFus shows superior performance compared to other text-mining resources

**Fig 4:**
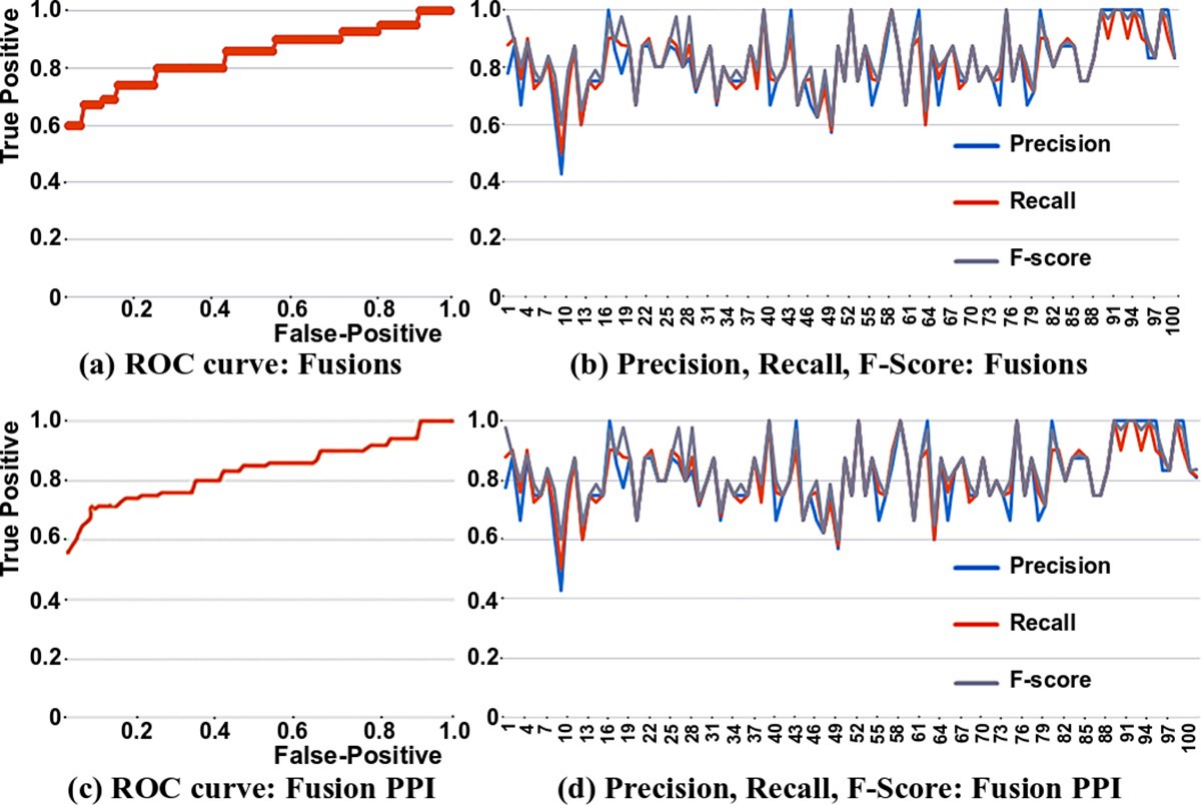
ROC curve for Naïve Bayes and accuracy. For fusions, the (a) ROC curve and (b) Precision, Recall, F-score; for fusion PPI (c) ROC curve and (d) Precision, Recall, F-score. Compared to full-text articles, prediction of a cancer type was more accurate for abstracts. This is because the size of feature space is too large for full-text articles. For text classification purposes, abstracts may yield better results than full-text scientific articles.

To demonstrate the added value of the ProtFus tool, we performed a direct comparison with existing services. ***Table 7*** represents the accuracy of ProtFus as compared to, ChimerDB-3.0 [19], FusionCancer [23] and FusionDB [36] resources. ChimerDB-3.0 chooses fusion gene candidate sentences from PubMed, which are further used for training a machine learning model. FusionCancer and FusionDB do not use text mining for fusion prediction. However, we used these datasets for resource-based comparisons of predicted fusion proteins. For the set of 1817 fusion proteins that were tested, the efficiency of our algorithm was about 92%, including the false positive rate, with respect to extracting fusion proteins and their PPIs from text. We compared ProtFus with other tools, according to Precision, Recall and F-score. We also found the Receiver Operating Characteristic (ROC) curves useful for quantitative representation of our method. ***Fig 4*** shows representative ROC curves generated in a typical experiment using ‘abstracts’ data. Compared to full-text articles, the extraction was better for abstracts. This is because the size of feature space is too large for full-text articles. For text classification purposes, abstracts may yield better results than full-text scientific articles. We also used various full-text journal corpus information for the purpose of evaluating our method’s performance over others [37]. Thus, text mining enables the inclusion of text-based data (unstructured data) in models that are subsequently for classification and clustering, and even anomaly detection. In our study, we used Bayesian learning to identify fusion proteins and their interactions. The effectiveness of ProtFus derives from the manner that it is used in specific cases. For example, the script can be updated to include different annotations associated with fusion proteins, which can be further used to study their properties.

## Conclusion

This study focused on investigating large-scale biomedical text classification downloaded from PubMed. We utilized classical text-mining and machine learning strategies, and also Big Data infrastructure to design and develop a distributed and scalable framework. This was applied to identify fusion proteins and their interactions for classifying information extracted from tens of thousands of abstracts and full-text articles with associated MeSH terms. The accuracy of predicting a cancer type by Naïve Bayes using the abstracts was 92%. In contrast, its accuracy using the 103,908 abstracts (for fusion proteins only), 90,639 full texts (for fusion proteins only), 185,606 abstracts (for fusion protein interactions) and 353,535 full texts (for fusion protein interactions) was 88%. This study demonstrates the potential for text mining of large-scale scientific articles using a novel Big Data infrastructure, with real-time updating from articles published daily. ProtFus can be extended to other areas of biomedical research to improve searches in multiple medical records and medical data mining approaches.

## Supporting information

S1 Table Root and relation tokens, Bibliography. (DOCX)

S2 Table Action tokens for fusion PPI. (DOCX)

S3 Table Synonyms for fusions, dictionary. (DOCX)

S4 Table Rulebase. (DOCX)

S5 Table Fusion tokens identified by ProtFus for 100 PubMed IDs. (DOCX)

S6 Table Fusion PPI tokens identified by ProtFus for 100 PubMed IDs. (DOCX)
