# Supplementary material for: ProtFus: A Comprehensive Method Characterizing Protein-Protein Interactions of Fusion Proteins
Source: PLoS Comput Biol. 2019 Aug 22;15(8):e1007239. doi: 10.1371/journal.pcbi.1007239 (PMC6705771; doi:10.1371/journal.pcbi.1007239)
Supplement: S1 Table — (DOCX) [file pcbi.1007239.s001.docx]

**Supplementary Table S1**

**ProtFus: A Comprehensive Method for Characterizing Protein-Protein Interactions of Fusion Proteins**

Somnath Tagore^1,3^, Alessandro Gorohovski^1^, Lars Juhl Jensen^2^ and Milana Frenkel-Morgenstern^1,*^

^1^ The Azrieli Faculty of Medicine, Bar-Ilan University, 8 Henrietta Szold St, Safed 13195, ISRAEL

^2^ Cellular Network Biology Group, The Novo Nordisk Foundation Center for Protein Research, University of Copenhagen, DENMARK

^3^ Present Address: Department of Systems Biology, Columbia University, New York, NY, 10032, USA.

*Corresponding Author E-mail: [milana.morgenstern@biu.ac.il](mailto:milana.morgenstern@biu.ac.il)

**Table S1: Root and relation tokens, Bibliography**

| **Root token** | **Relation tokens** |
| --- | --- |
| activate | activates, activating, activator |
| block | blocks, blocking, blocked, blocks in, blocks with |
| chimera | chimeric, chimeric gene, chimeric genes, chimeric transcript |
| depend | dependent, depends on, depends to, depending on |
| domain | domains |
| express | expression, expressed in, expressed with, expresses in |
| family |  |
| fusion | fusions, fusion transcript, fusion transcripts, fusion protein, fusion proteins, fusion gene, fusion genes |
| gene | genes, gene fusion |
| interaction | interactions, interactions with |
| protein | proteins |
| reduce | reduced, reduced form |
| residue | residues |
| transcript | transcripts |
