# Supplementary material for: ProtFus: A Comprehensive Method Characterizing Protein-Protein Interactions of Fusion Proteins
Source: PLoS Comput Biol. 2019 Aug 22;15(8):e1007239. doi: 10.1371/journal.pcbi.1007239 (PMC6705771; doi:10.1371/journal.pcbi.1007239)
Supplement: S2 Table — (DOCX) [file pcbi.1007239.s002.docx]

**Supplementary Table S2**

**ProtFus: A Comprehensive Method for Characterizing Protein-Protein Interactions of Fusion Proteins**

Somnath Tagore^1,3^, Alessandro Gorohovski^1^, Lars Juhl Jensen^2^ and Milana Frenkel-Morgenstern^1,*^

^1^ The Azrieli Faculty of Medicine, Bar-Ilan University, 8 Henrietta Szold St, Safed 13195, ISRAEL

^2^ Cellular Network Biology Group, The Novo Nordisk Foundation Center for Protein Research, University of Copenhagen, DENMARK

^3^ Present Address: Department of Systems Biology, Columbia University, New York, NY, 10032, USA.

*Corresponding Author E-mail: [milana.morgenstern@biu.ac.il](mailto:milana.morgenstern@biu.ac.il)

**Table S2: Action Tokens for Fusion PPIs**

| **Root token** | **Relation tokens** |
| --- | --- |
| Abolish | abolish, abolishes, abolished, abolishing |
| Accelerate | accelerate, accelerates, accelerated, accelerating |
| Acceptor | acceptor |
| Accumulate | accumulate, accumulates, accumulated, accumulating, accumulation |
| Acetylate | acetylate, acetylates, acetylated, acetylating, acetylation |
| Activate | activate, activates, activated, activating, activation, activator |
| Affect | affect, affects, affected, affecting |
| Alter | alter, alters, altered, altering, alteration |
| Amplify | amplify, amplifies, amplified, amplifying, amplification |
| Apoptosis | apoptosis |
| Assemble | assemble, assembles, assembled, assembling |
| Associate | associate, associates, associated, associating, association |
| Attach | attach, attaches, attached, attaching, attachment |
| Attack | attack, attacks, attacked, attacking |
| Bind | bind, binds, bound, binding |
| Block | block, blocks, blocked, blocking |
| Carbamoylate | carbamoylate, carbamoylates, carbamoylated, carbamoylating, carbamoylation |
| Carboxylate | carboxylate, carboxylates, carboxylated, carboxylating, carboxylation |
| Catalyze | catalyze, catalyzes, catalyzed, catalyzing |
| Cleave | cleave, cleaves, cleaved, cleaving |
| Co-immunoprecipitate | co-immunoprecipitate, co-immunoprecipitates,  co-immunoprecipitated, co-immunoprecipitating,  co-immunoprecipitation, co-immunoprecipitations |
| Compare | compared, comparison, compared to |
| Complex | complex, complexes, complexed, complexing, complexation |
| Conjugate | conjugate, conjugates, conjugated, conjugating, conjugation |
| Contact | contact, contacts, contacted, contacting |
| Couple | coupled, coupled with, coupled to |
| Covalent | covalent link, covalently linked to |
| Deaccetylate | deaccetylate, deaccetylates, deaccetylated, deaccetylating, deaccetylation |
| Deaminate | deaminate, deaminates, deaminated, deaminating, deamination |
| Decarboxylate | decarboxylate, decarboxylates, decarboxylated, decarboxylating, decarboxylation |
| Decrease | decrease, decreases, decreased, decreasing |
| Dehydrate | dehydrate, dehydrates, dehydrated, dehydrating, dehydration |
| Dehydrogenate | dehydrogenate, dehydrogenates, dehydrogenated, dehydrogenating, dehydrogenation |
| Demethylate | demethylate, demethylates, demethylated, demethylating, demethylation |
| Dephosphorylate | dephosphorylate, dephosphorylates, dephosphorylated, dephosphorylating, dephosphorylation |
| Deplete | deplete, depletes, depleted, depleting, depletion |
| Disassemble | disassemble, disassembles, disassembled, disassembling |
| Discharge | discharge, discharges, discharged, discharging |
| Dock | dock, docks, docked, docking |
| Down-regulate | down-regulate, down-regulates, down-regulated, down-regulating, down-regulation |
| Downregulate | downregulate, downregulates, downregulated, downregulating, downregulation |
| Elevate | elevate, elevates, elevated, elevating, elevation |
| Enhance | enhance, enhances, enhanced, enhancing |
| Express | express, expresses, expressed, expressing, expression, express as |
| Formylate | formylate, formylates, formylated, formylating, formylation |
| Fusion | fusions, fusion proteins |
| Glycosylate | glycosylate, glycosylates, glycosylated, glycosylating, glycosylation |
| Hasten | hasten, hastens, hastening, hastened |
| Heterodimerize | heterodimerize, heterodimerizes, heterodimerizing, heterodimerized, heterodimerization, heterodimer, heterodimers |
| Homodimerize | homodimerize, homodimerizes, homodimerizing, homodimerized, homodimerization, homodimer, homodimers |
| Hydrolyse | hydrolyse, hydrolyses, hydrolysing, hydrolysed, hydrolysis |
| Inactivate | inactivate, inactivates, inactivated, inactivating, inactivation |
| Incite | incite, incites, incited, inciting |
| Induce | induce, induces, induced, inducing, induction |
| Infect | infect, infects, infected, infecting |
| Influence | influence, influences, influencing, influenced |
| Inhibit | inhibit, inhibits, inhibited, inhibiting, inhibition, inhibitors |
| Initiate | initiate, initiates, initiated, initiating, initiation |
| Interact | interact, interacts, interacts, interacting, interaction |
| Impair | impair, impairs, impaired, impairing |
| Isomerize | isomerize, isomerizes, isomerized, isomerizing, isomerization |
| Ligate | ligate, ligates, ligated, ligating, ligation |
| Mediate | mediate, mediates, mediated, mediating |
| Methylate | methylate, methylates, methylated, methylating, methylation |
| Modify | modify, modifies, modified, modifying, modification |
| Modulate | modulate, modulates, modulating, modulated |
| Myogenesis | myogenesis |
| Overexpress | overexpress, overexpresses, overexpressed, overexpressing, overexpression |
| Oxidize | oxidize, oxidizes, oxidized, oxidizing, oxidation |
| Pair | pair, pairs, paired, paring |
| Participate | participate, participates, participated, participating, participation |
| Peroxidize | peroxidize, peroxidizes, peroxidized, peroxidizing, peroxidation |
| Phosphorylate | phosphorylate, phosphorylates, phosphorylated, phosphorylating, phosphorylation |
| Prevent | prevent, prevents, prevented, preventing |
| Produce | produce, produces, produced, producing, production |
| Promote | promote, promotes, promoted, promoting, promotion |
| Protein | Protein, proteins, protein-protein, PPI |
| React | react, reacts, reacted, reacting, reaction |
| Recognize | recognize, recognizes, recognized, recognizing, recognition |
| Recruit | recruit, recruits, recruited, recruiting |
| Regulate | regulate, regulates, regulated, regulating, regulation |
| Replace | replace, replaces, replaced, replacing |
| Repress | repress, represses, repressed, repressing, repression |
| Severe | severe, severed, severing |
| Split | split, splitting |
| Stimulate | stimulate, stimulates, stimulated, stimulating, stimulation, stimulator |
| Substitute | substitute, substitutes, substituted, substituting, substitution |
| Suppress | suppress, suppresses, suppressed, suppressing, suppression |
| Tether | tether, tethers, tethered, tethering |
| Transactivate | transactivate, transactivates, transactivated, transactivating, transactivation, transactivator |
| Transaminate | transaminate, transaminates, transaminated, transaminating, transamination |
| Ubiquitinate | ubiquitinate, ubiquitinates, ubiquitinated, ubiquitinating, ubiquitination |
| Upregulate | upregulate, upregulates, upregulated, upregulating, upregulation, upregulator |
| Up-regulate | up-regulate, up-regulates, up-regulated, up-regulating, up-regulation, up-regulator |
