# Supplementary material for: ProtFus: A Comprehensive Method Characterizing Protein-Protein Interactions of Fusion Proteins
Source: PLoS Comput Biol. 2019 Aug 22;15(8):e1007239. doi: 10.1371/journal.pcbi.1007239 (PMC6705771; doi:10.1371/journal.pcbi.1007239)
Supplement: S3 Table — (DOCX) [file pcbi.1007239.s003.docx]

**Supplementary Table S3**

**ProtFus: A Comprehensive Method for Characterizing Protein-Protein Interactions of Fusion Proteins**

Somnath Tagore^1,3^, Alessandro Gorohovski^1^, Lars Juhl Jensen^2^ and Milana Frenkel-Morgenstern^1,*^

^1^ The Azrieli Faculty of Medicine, Bar-Ilan University, 8 Henrietta Szold St, Safed 13195, ISRAEL

^2^ Cellular Network Biology Group, The Novo Nordisk Foundation Center for Protein Research, University of Copenhagen, DENMARK

^3^ Present Address: Department of Systems Biology, Columbia University, New York, NY, 10032, USA.

*Corresponding Author E-mail: [milana.morgenstern@biu.ac.il](mailto:milana.morgenstern@biu.ac.il)

**Table S3: Synonyms for Fusions, Dictionary**

| **Fusion proteins** | **Synonyms** | **Alternate representations** |
| --- | --- | --- |
| EWS-FLI1 | SH2D1B EAT2 , ELK1, PDGFC SCDGF, ETV2 ER71,ETSRP71 | ews fli1, EWSR1 EWS, EWSR1/FLI1, EWS FLI-1 |
| Fc-containing | NA | NA |
| motif-GST | NA | NA |
| EPO-Fc | NA | NA |
| VpHsf-GFP | NA | NA |
| GLP-1 | NA | NA |
| GLP-1 | NA | NA |
| GFP-SlGGB1 | NA | NA |
| receptor-Fc | NA | NA |
| LSL-tagged | NA | NA |
| LSL-tagged | NA | NA |
| Ag85B-ESAT6 | NA | NA |
| Rep/VP | rep VP0041, rep VP02_18990 | Rep-VP |
| Rep/VP | NA | NA |
| NS1-epitope | NA | NA |
| DISC1-Boymaw | NA | NA |
| DISC1-Boymaw | NA | NA |
| AML1/ETO | RUNX1 AML1,CBFA2, RUNX1T1 AML1T1,CBFA2T1,CDR,ETO,MTG8, TCF12 BHLHB20,HEB,HTF4, SON C21orf50,DBP5,KIAA1019,NREBP,HSPC310, CBFA2T3 MTG16,MTGR2,ZMYND4 | AML1-ETO |
| AcrB-AcrA | NA | NA |
| Antibody-cytokine | NA | NA |
| SRRP1-GFP | NA | NA |
| E3-ubiquitin | NA | NA |
| PD-LIg | NA | NA |
| PD-L1Ig | NA | NA |
| receptor-Fc | NA | NA |
| NPM-ALK | ZC3HC1 NIPA,HSPC216, PPID CYP40,CYPD, SFPQ PSF | NPM/ALK |
| GluA2-GluK | NA | NA |
| F-specific | NA | NA |
| HN-specific | NA | NA |
| His-SUMO | NA | NA |
| VP2-VP3 | NA | NA |
| VP2-VP3 | NA | NA |
| his-tag | NA | NA |
| TMPRSS2-ERG | ERG | TMPRSS2/ERG |
| RET/PTC | RET CDHF12,CDHR16,PTC,RET51, TRIM24 RNF82,TIF1,TIF1A, TRIM33 KIAA1113,RFG7,TIF1G, NCOA4 ARA70,ELE1,RFG, MAPK15 ERK7,ERK8, SH2B1 KIAA1299,SH2B, PDLIM7 ENIGMA, RET-ELE1, ELE1-RET | RET/PTC2, RET-PTC |
| BMP7-BMP2 | BMPR2 PPH1, Bmpr2 | BMPP7/BMP2 |
| MAP-1 | NA | NA |
| PROTEIN-ES1 | NA | NA |
| RPS6-AtHD2B | NA | NA |
| thioredoxin-tagged | NA | NA |
| NKp80-Fc | NA | NA |
| NKp80-Fc | NA | NA |
| BCR-ABL1 | SETBP1 KIAA0437, BTK, Bach2, ZNFN1A1 IKZF1, | BCR/ABL1 |
| BCR-ABL1 | NA | NA |
| Vpr-IN | vpr, VPRBP DCAF1,KIAA0800,RIP, | Vpr/IN |
| protein-NCX1 | NA | NA |
| hepcidin-thioredoxin | NA | NA |
| EML4-ALK | EML1 EMAP1,EMAPL,EMAPL1, EML2 EMAP2,EMAPL2 | EML4/ALK |
| NAB2-STAT6 | NA | NA |
| NAB2-STAT6 | NA | NA |
| NAB2-STAT6 | NA | NA |
| NAB2-STAT6 | NA | NA |
| LDHC-RFP | NA | NA |
| protein-nitroreductase | NA | NA |
| antibody/Fc | NA | NA |
| BCR-ABL | NA | NA |
| catalase-lipoxygenase | NA | NA |
| catalase-lipoxygenase | NA | NA |
| KIR-Fc | NA | NA |
| MYB-NFIB | MYB/NFIB | MYB/NFIB |
| IFNalpha1-THYalpha1 | NA | NA |
| poIFNalpha1-THYalpha1 | NA | NA |
| poIFNalpha1-THYalpha1 | NA | NA |
| envelope-NS1 | NA | NA |
| Flag-tagged | NA | NA |
| Flag-tagged | NA | NA |
| MIIA-Strep | NA | NA |
| hsp70-p24 | NA | NA |
| EGFP-HttQ52 | NA | NA |
| AdoMetDC-SpdSyn | NA | NA |
| protein-tagged | NA | NA |
| signaling-enhanced | NA | NA |
| BCR-ABL | NA | NA |
| CD19-specific | NA | NA |
| Osteocalcin-fibronectin | NA | NA |
| antibody-cytokine | NA | NA |
| SP-target | NA | NA |
| SP-LacA | NA | NA |
| cell-permeable | NA | NA |
| HA-Fc | NA | NA |
| GFP-NACC1 | NA | NA |
| flagellin-PAc | NA | NA |
| EML4-ALK | NA | NA |
| IL-2 | NA | NA |
| cell-permeable | NA | NA |
| B7-specific | NA | NA |
| translocation-associated | NA | NA |
| PgMYB1-mGFP5 | NA | NA |
| CTCR3-MAML2 | NA | NA |
| CCDC6-RET | RET CDHF12,CDHR16,PTC,RET51, CCDC6-RETc, | CCDC6/RETc, |
| CCDC6-RET | NA | NA |
| CCR5/CCR2b | NA | NA |
| Alpha-TFEB | NA | NA |
| peptide-Fc | NA | NA |
| X-MLL | NA | NA |
| RUNX1-RUNX1T1 | RUNX1 RUNX1T1 | RUNX1/RUNX1T1 |
| androgen-regulated | NA | NA |
| mucin-type | NA | NA |
| OSCAR-Fc | NA | NA |
| TREM2-Fc | NA | NA |
| TREM2-Fc | NA | NA |
| Mer/Fc | NA | NA |
| FP-tubulin | NA | NA |
| FP-Tub1 | NA | NA |
| FUS-KLF17 | NA | NA |
| EWS-FLI1 | NA | NA |
| Antibody-cytokine | NA | NA |
| CBFbeta-SMMHC | NA | NA |
| IL-2 | NA | NA |
| 2-STAT6 | NA | NA |
| gamma-gliadin | NA | NA |
| TNF-induced | NA | NA |
| TTP-susceptible | NA | NA |
| r-Cpae | NA | NA |
| bio-functional | NA | NA |
| PutAPX-GFP | NA | NA |
| ZZ-AP | NA | NA |
| TACI-Ig | NA | NA |
| TPM3-ALK | NA | NA |
| NAB2-STAT6 | NA | NA |
| PSF-TFE3 | TFE3 BHLHE33 | PSF/TFE3 |
| EML4-ALK | NA | NA |
| EML4-ALK | NA | NA |
| EML4-ALK | NA | NA |
| Rec11-Rec10 | NA | NA |
| S-transferase | NA | NA |
| GFP-HNF1alpha | NA | NA |
| CD30-Immunoglobulin | NA | NA |
| PML-RARa | PML MYL,PP8675,RNF71,TRIM19, PRAM1, UBE2I UBC9,UBCE9, RARA NR1B1, SUMO1 SMT3C,SMT3H3,UBL1,OK/SW-cl.43, SUV39H1 KMT1A,SUV39H, PIAS2 PIASX, CSNK2A1 CK2A1, SUMO2 SMT3B,SMT3H2, RARA | PML/RARA |
| BCR-ABL1 | NA | NA |
| BCR-ABL1 | NA | NA |
| BCR-ABL1 | NA | NA |
| TPM3-NTRK1 | NA | NA |
| neoplasia-associated | NA | NA |
| anti-CD19 | NA | NA |
| PmERP15-EGFP | NA | NA |
| above-mentioned | NA | NA |
| TNS3-MAP3K3 | ZFPM2-ELF5 | TNS3/MAP3K3 |
| ZFPM2-ELF5 | MAP3K3-TNS3, TNS3-MAP3K3 | ZFPM2/ELF5 |
| TAT-Nanog | NA | NA |
| TACI-Ig | NA | NA |
| IL-2 | NA | NA |
| IL-2 | NA | NA |
| IL-2 | NA | NA |
| EWSR1-WT1 | NA | NA |
| M3-T4L | NA | NA |
| mouse-porcine | NA | NA |
| antibody-cytokine | NA | NA |
| antibody-cytokine | NA | NA |
| antibody-cytokine | NA | NA |
| cell-cell | NA | NA |
| GST-CTD | NA | NA |
| GPR84-Gialpha | NA | NA |
| bi-functional | NA | NA |
| NCOA4/RET | NA | NA |
| PAX3-FOXO1 | FGFR4 JTK2,TKF, PAX3 HUP2, FAM193B IRIZIO,KIAA1931 | PAX3/FOXO1 |
| MDR1-mApple | NA | NA |
| EGFP-rab11a | NA | NA |
| promoter-reporter | NA | NA |
| JAGGED2-Fc | NA | NA |
| SF301-mCherry | NA | NA |
| synthase/phosphatase | NA | NA |
| GPC3-targeted | NA | NA |
| FN1-FGFR1 | NA | NA |
| NKp30-Fc | NA | NA |
| BCR-ABL | NA | NA |
| N-terminal | NA | NA |
| TCF3-PBX1 | TCF3 BHLHB21,E2A,ITF1, PBX1 PRL, ANKS1B | TCF3/PBX1 |
| L20h-Ts3 | L20h-Ts3 | L20h/Ts3 |
| L20h-Ts3 | NA | NA |
| TMPRSS2-ERG | ERG | TMPRSS2/ERG |
| TMPRSS2-ERG | NA | NA |
| TMPRSS2-ERG | NA | NA |
| TMPRSS2-ERG | NA | NA |
| GFP-PGRMC2 | NA | NA |
| LapA-GFP | NA | NA |
| TMPRSS2-ERG | NA | NA |
| gamete-specific | NA | NA |
| PR8/WSN | NA | NA |
| EWSR1-related | NA | NA |
| EWSR1-PBX3 | NA | NA |
| EWSR1-PBX3 | NA | NA |
| EWSR1-related | NA | NA |
| EWSR1-CREB3L1 | NA | NA |
| FUS-CREB3L2 | CREB3L2 BBF2H7 | HUS/CREB3L1 |
| GFP-VirB11 | NA | NA |
| 18-kDa | NA | NA |
| MYB-NFIB | MYB/NFIB | MYB/NFIB |
| MYB-NFIB | NA | NA |
| MYB-NFIB | NA | NA |
| NKG2D-IgG1 | NA | NA |
| TAT-gelonin | NA | NA |
| F8-IL4 | NA | NA |
| BCOR-CCNB3 | NA | NA |
| ICAM1-Fc | NA | NA |
| EML4-ALK | NA | NA |
| mouse/human | NA | NA |
| NOTCH3-Fc | NA | NA |
| G-C5a | NA | NA |
| BclS-GFP | NA | NA |
| FH/Fc | NA | NA |
| FH/Fc | NA | NA |
| SNAP-tag | NA | NA |
| SNAP-tagged | NA | NA |
| single-chain | NA | NA |
| in-frame | NA | NA |
| EstA-autotransporter | NA | NA |
| YFP-sarcomeric | NA | NA |
| MECT1-MAML2 | CRTC1 KIAA0616,MECT1,TORC1,WAMTP1 | MECT1/MAML2 |
| DOX-CYP | NA | NA |
| GP16-EGFP | NA | NA |
| CD19-specific | NA | NA |
| CD9-GFP | NA | NA |
| Pvs25-PvCSP | NA | NA |
| TMPRSS2-ERG | NA | NA |
| scFv425-sTRAIL | NA | NA |
| Hv1a/GNA | NA | NA |
| Pl1a/GNA | NA | NA |
| Hv1a/GNA | NA | NA |
| protein-hPXR | NA | NA |
| PABD-YFP | NA | NA |
| FC5-Fc | NA | NA |
| luciferase-PAWP | NA | NA |
| Rab3A/Rab22A | TBC1D10B FP2461, Tbc1d10b | Rab3A-Rab22A |
| single-chain | NA | NA |
| GMCSF-NAg | NA | NA |
| WWTR1-FOSB | NA | NA |
| ABCD2-EGFP | NA | NA |
| FAM131B-BRAF | NA | NA |
| NPM-RAR | NA | NA |
| Gag-Pol | NA | NA |
| EWSR1-FLI1 | EWSR1/FLI1 | EWSR1/FLI1 |
| Fc-tagged | NA | NA |
| EWS/ETS | EWSR1 EWS , FEV PET1 | EWS-ETS |
| PrgI-SipD | NA | NA |
| beta-lactamase | NA | NA |
| DT-IL3 | NA | NA |
| DDX3X-PRKD1 | NA | NA |
| ARID1A-PRKD1 | NA | NA |
| LMP1/CD40 | NA | NA |
| agonist/antagonist | NA | NA |
| protein-coding | NA | NA |
| NP-CD40L | NA | NA |
| 6xHis-TdsPLA2III | NA | NA |
| SUMO-Tpz1 | NA | NA |
| Azu-P450 | NA | NA |
| AML1/ETO | RUNX1 AML1,CBFA2, RUNX1T1 AML1T1,CBFA2T1,CDR,ETO,MTG8,TCF12 BHLHB20,HEB,HTF4 , SON C21orf50,DBP5,KIAA1019,NREBP,HSPC310, CBFA2T3 MTG16,MTGR2,ZMYND4 , Cbfa2t3 Cbfa2t3h,Mtgr2 , Usp18 Ubp43 , pir | AML1-ETO |
| GFP-scFv | NA | NA |
| C-terminal | NA | NA |
| PML-RARA | NA | NA |
| PML-RARA | NA | NA |
| PML-RARalpha | PML MYL,PP8675,RNF71,TRIM19 , RARA NR1B1 , UBE2I UBC9,UBCE9 , Trim24 Tif1,Tif1a | PML/RARalpha |
| EWSR1-FEV | NA | NA |
| EWSR1-FLI1 | EWSR1/FLI1 | EWSR1/FLI1 |
| EML4-ALK | EML1 EMAP1,EMAPL,EMAPL1, EML2 EMAP2,EMAPL2 | EML4/ALK , EML4-ALK, |
| IgG-IDS | NA | NA |
| HIRMAb-IDS | NA | NA |
| HIRMAb-IDS | NA | NA |
| HIRMAb-IDS | NA | NA |
| HIRMAb-IDS | NA | NA |
| ERV-Flt3 | NA | NA |
| scFvBaP1-SUMO | NA | NA |
| receptor-Galpha | NA | NA |
| pirB-cry2Aa | NA | NA |
| L4-L5 | NA | NA |
| SLK-LacZ | NA | NA |
| SLK-LacZ | NA | NA |
| SLK-LacZ | NA | NA |
| KRT5/KRT8 | NA | NA |
| cancer-specific | NA | NA |
| BCR-ABL | NA | NA |
| tumor-specific | NA | NA |
| MICA-Fc | NA | NA |
| Trx-hGH | NA | NA |
| FGFR3-TACC3 | NA | NA |
| FGFR3-TACC3 | NA | NA |
| GST-TvCyP1 | NA | NA |
| B-C2 | NA | NA |
| REST-VP16 | NA | NA |
| Trx-hCTRP1 | NA | NA |
| EWS-FLI1 | NA | NA |
| EWS/FLI | NA | NA |
| EaF82a-sGFP | NA | NA |
| c-myc | NA | NA |
| antibody-cytokine | NA | NA |
| F8-IFNgamma | NA | NA |
| antibody-cytokine | NA | NA |
| FGFR3-TACC3 | NA | NA |
| CD19-specific | NA | NA |
| Fla-L2 | NA | NA |
| TMPRSS2-ERG | NA | NA |
| TMPRSS2-ERG | NA | NA |
| His-tagged | NA | NA |
| NPM-ALK | NA | NA |
| TRPC6-V5 | NA | NA |
| JAZF1-SUZ12 | SUZ12 CHET9,JJAZ1,KIAA0160 , JAZF1 TIP27,ZNF802 | JAZF1/SUZ12 |
| EGFP-CPP | NA | NA |
| EWSR1-NR4A3 | NA | NA |
| TAF15-NR4A3 | NA | NA |
| TCF12-NR4A3 | NA | NA |
| TAT-gelonin | NA | NA |
| TRIM24-BRAF | Trim24 Tif1,Tif1a | TRIM24/BRAF |
| Cell-cell | NA | NA |
| cell-cell | NA | NA |
| GFP-STRS | NA | NA |
| IL13Ralpha2-targeted | NA | NA |
| CIC-DUX4 | CIC/DUX4 | CIC/DUX4 |
| LAMTOR1-PRKCD | NA | NA |
