# Supplementary material for: ProtFus: A Comprehensive Method Characterizing Protein-Protein Interactions of Fusion Proteins
Source: PLoS Comput Biol. 2019 Aug 22;15(8):e1007239. doi: 10.1371/journal.pcbi.1007239 (PMC6705771; doi:10.1371/journal.pcbi.1007239)
Supplement: S4 Table — (DOCX) [file pcbi.1007239.s004.docx]

**Supplementary Table S4**

**ProtFus: A Comprehensive Method for Characterizing Protein-Protein Interactions of Fusion Proteins**

Somnath Tagore^1,3^, Alessandro Gorohovski^1^, Lars Juhl Jensen^2^ and Milana Frenkel-Morgenstern^1,*^

^1^ The Azrieli Faculty of Medicine, Bar-Ilan University, 8 Henrietta Szold St, Safed 13195, ISRAEL

^2^ Cellular Network Biology Group, The Novo Nordisk Foundation Center for Protein Research, University of Copenhagen, DENMARK

^3^ Present Address: Department of Systems Biology, Columbia University, New York, NY, 10032, USA.

*Corresponding Author E-mail: [milana.morgenstern@biu.ac.il](mailto:milana.morgenstern@biu.ac.il)

**Table S4: Rulebase**

| **Description** | **Rule** | **Reg Ex** |
| --- | --- | --- |
| Starting with space | Should follow with a letter/token | \s\w+ |
| Tokens separated with a dash | Should be considered a fusion token | \w+(\-)\w+ |
| Tokens separated with a colon | Should be considered a fusion token | \w+(\:)\w+ |
| Tokens separated with a front slash | Should be considered a fusion token | \w+(\/)\w+ |
| Tokens separated by a space | Should follow with a letter/token | \w+\s+w+ |
| Tokens with fusion word occurrence | Should be separated by space/tokens | \s\w+\s(('fusion\|fusions\|fusion genes\|gene fusion\|fusion protein\|fusion transcripts')\s |
| Any Greek letter in the middle | Should be considered a non-English token | \w+ [aßYÖE ] \w+ |
| Tokens with chimeric word occurrence | Should be separated by space/tokens | \s\w+\s('chimeric\|chimeric transcript\|chimeric gene')\s |
| Protein name with a number in the middle | Should be considered an alpha-neumeric token | \w+[0-9]\w+ |
| Tokens with transcript word occurrence | Should be separated by space/tokens | \s\w+\s('transcripts')\s |
| Tokens with chimeric word occurrence followed by an adjective or vice-versa | Should be considered a fusion token if an adjective has a dash/colon/front slash | (\s(chimera\|chimeric\|chimeric gene\|chimeric transcript )\s\w+(\-\|\:\|\/)\w+\s) OR (\s\w+(\-\|\:\|\/)\w+\s(chimera\|chimeric\|chimeric gene\|chimeric transcript )\s) |
| Last character is a dash | Part of q fusion protein | \w+ - |
| Tokens with fusion word occurrence followed by an adjective or vice-versa | Should be considered a fusion token if an adjective has a dash/colon/front slash | (\s(fusion\|fusions\|fusion transcript\|fusion transcripts\|fusion proteins\|fusion genes )\s\w+(\-\|\:\|\/)\w+\s) OR (\s\w+(\-\|\:\|\/)\w+\s(fusion\|fusions\|fusion transcript\|fusion transcripts\|fusion proteins\|fusion genes )\s) |
| First character is a dash | Part of a fusion protein | -\w+ |
| Tokens with depend word occurrence preceded and succeeded by an adjective token | Should be considered an interaction | \s\w+(\-\|\:\|\/)\w+\s(dependent\|depends on\|independent\|depends to\|depending on )\s\w+(\-\|\:\|\/)\w+\s) |
| All letters are in uppercase | N/A | [A-Z]+ |
| Tokens with express word occurrence preceded and succeeded by an adjective token | Should be considered an interaction | \s\w+(\-\|\:\|\/)\w+\s(express\|expression\|expressed in\|expressed with\|expresses in )\s\w+(\-\|\:\|\/)\w+\s) |
| Any natural number | N/A | [0-9]+ |
| Tokens with interact word occurrence preceded and succeeded by an adjective token | Should be considered an interaction | \s\w+(\-\|\:\|\/)\w+\s(interact\|induce\|initiate\|modulate\|produce\|incite )\s\w+(\-\|\:\|\/)\w+\s) |
| Tokens with negative word occurrence preceded and succeeded by an adjective token | Should be considered an interaction | \s\w+(\-\|\:\|\/)\w+\s(dephosphorylate\|decarboxylate\| demethylate\|deaccetylate\|deaminate\|dehydrogenate)\s\w+(\-\|\:\|\/)\w+\s) |
| First letter is in uppercase | N/A | [A-Z]\w+ |
| Tokens with positive word occurrence preceded and succeeded by an adjective token | Should be considered an interaction | \s\w+(\-\|\:\|\/)\w+\s(phosphorylate\|phosphorylated\|phosphorylation\|acetylate\|acetylated\|acetylation\|carboxylate\|carbamoylate\|dephosphorylate\|decarboxylate\|methylate\|formylate\|glycosylate\|ubiquitinate\|transaminate)\s\w+(\-\|\:\|\/)\w+\s) |
| Any Roman letter in the middle | N/A | \w+ [IVXDLCM]+ \w+ |
| Combination of letters and numbers. First character is a letter. | N/A | \w+ [A-Za-z] \w+ [0-9]\w+ |
| Any Roman letter | N/A | [IVXDLCM]+ |
| Tokens with process word occurrence preceded and succeeded by an adjective token | Should be considered an interaction | \s\w+(\-\|\:\|\/)\w+\s(enhance\|enhanced\|enhancing\|enhances\|amplify\|amplifies\|elevate\|express\|promote\|influence\|react\|mediate)\s\w+(\-\|\:\|\/)\w+\s) |
| Mixture of uppercase and lowercase letters | N/A | [A-Za-z]+ |
| Tokens with increased activity word occurrence preceded and succeeded by an adjective token | Should be considered an interaction | \s\w+(\-\|\:\|\/)\w+\s(activate\|activates\|activating\|activator\|accelerate\|accelerating\|affect\|stimulate\|regulate)\s\w+(\-\|\:\|\/)\w+\s) |
| Two-digit numbers | N/A | [0-9][0-9] |
| Tokens with decreased activity word occurrence preceded and succeeded by an adjective token | Should be considered an interaction | \s\w+(\-\|\:\|\/)\w+\s(block\|blocks\|blocking\|blocks in\|blocks with\|blocked\|attacks\|attacked\|abolish)\s\w+(\-\|\:\|\/)\w+\s) |
| Combination of letters and numbers. First character is a number. | N/A | \w+ [0-9] \w+ [A-Za-z]\w+ |
| Tokens with breakdown event word occurrence preceded and succeeded by an adjective token | Should be considered an interaction | \s\w+(\-\|\:\|\/)\w+\s(catalyze\|cleave\|dissassemble)\s\w+(\-\|\:\|\/)\w+\s) |
| First letter is in uppercase. Second letter is in lowercase. | N/A | [A-Z][a-z]\w+ |
| Tokens with negation event word occurrence preceded and succeeded by an adjective token | Should be considered an interaction | \s\w+(\-\|\:\|\/)\w+\s(alter\|decrease\|deplete\|discharge\|downregulate\|inactivate\|inhibit\|impair\|modify\|prevent\|repress\|suppress\|tether)\s\w+(\-\|\:\|\/)\w+\s) |
| Ranges from 0 to 9 | N/A | [0-9] |
| Tokens with drill-down event word occurrence preceded and succeeded by an adjective token | Should be considered an interaction | \s\w+(\-\|\:\|\/)\w+\s(hydrolyse\|isomerize\|ligate\|oxidize\|peroxidize\|transactivate\|heterodimerize\|homodimerize\|split)\s\w+(\-\|\:\|\/)\w+\s) |
| Any Greek letter | N/A | \w+ [aßYÖE ] |
| Tokens with roll-up event word occurrence preceded and succeeded by an adjective token | Should be considered an interaction | \s\w+(\-\|\:\|\/)\w+\s(assemble\|acceptor\|accumulate\|associate\|attach\|bind\|complex\|conjugate)\s\w+(\-\|\:\|\/)\w+\s) |
