# Supplementary material for: ProtFus: A Comprehensive Method Characterizing Protein-Protein Interactions of Fusion Proteins
Source: PLoS Comput Biol. 2019 Aug 22;15(8):e1007239. doi: 10.1371/journal.pcbi.1007239 (PMC6705771; doi:10.1371/journal.pcbi.1007239)
Supplement: S5 Table — (DOCX) [file pcbi.1007239.s005.docx]

**Supplementary Table S5**

**ProtFus: A Comprehensive Method for Characterizing Protein-Protein Interactions of Fusion Proteins**

Somnath Tagore^1,3^, Alessandro Gorohovski^1^, Lars Juhl Jensen^2^ and Milana Frenkel-Morgenstern^1,*^

^1^ The Azrieli Faculty of Medicine, Bar-Ilan University, 8 Henrietta Szold St, Safed 13195, ISRAEL

^2^ Cellular Network Biology Group, The Novo Nordisk Foundation Center for Protein Research, University of Copenhagen, DENMARK

^3^ Present Address: Department of Systems Biology, Columbia University, New York, NY, 10032, USA.

*Corresponding Author E-mail: [milana.morgenstern@biu.ac.il](mailto:milana.morgenstern@biu.ac.il)

**Table S5: Fusion tokens identified by ProtFus for 100 PubMed IDs**

| Fusion_ID | gene1 | gene2 | pubmed | info |
| --- | --- | --- | --- | --- |
| F000001 | EZR | ROS1 | 24186139 | CD74-ROS1 and EZR-ROS1 fusions were significantly associated with at least focal globular immunoreactivity and plasma membranous accentuation, respectively, and these patterns were specific to ROS1-rearranged cases. |
| F000002 | ACSL3 | ETV1 | 22575261 | Subsequent molecular studies confirmed the presence of an associated ACTB-GLI1 fusion transcript. |
| F000003 | ACTB | GLI1 | 24561789 | Size and location of functional domains of the MLL wt, ACTN4 wt, and of the MLL-ACTN4 fusion protein. |
| F000004 | ACTN4 | MLL | 23762276 | Seven of the twelve fusion transcripts were classified as before endoreduplication; two, CTCF-SCUBE2 and BC041478-EXOSC10 were classified later. AGPAT5-MCPH1 and SUSD1-ROD1/PTBP3 and KLK5-CDH23 were undetermined, as their allelic copy number could not be resolved by array CGH or FISH. These structural rearrangements gave rise to at least twelve expressed fusion transcripts, confirmed by RT-PCR and Sanger sequencing: RGS22-SYCP1, CTAGE5-SIP1, PLXND1-TMCC1, SEC22B-NOTCH2, KLK5-CDH23, BC041478-EXOSC10, AGPAT5-MCPH1, SUSD1-ROD1/PTBP3, SGK1-SLC2A12, RHOJ-SYNE2, PUM1-TRERF1 and CTCF-SCUBE2 |
| F000005 | AFF1 | MLL | 20613748 | The SCL45A3-BRAF fusion mRNA encodes a 329–amino acid protein that comprises only a C-terminal fragment of BRAF. By contrast, the ESRP1-RAF2 and AGTRAP-BRAF fusions encode proteins with substantial contribution of N-terminal sequences from the RAF fusion partner. |
| F000006 | AGPAT5 | MCPH1 | 23543667 | In addition, common oncogenic fusions of RET and NTRK1 as well as PAX8/PPARγ and AKAP9-BRAF were also assessed by RT-PCR. |
| F000007 | AGTRAP | BRAF | 11310834 | The hybrid MSN-ALK protein had a molecular weight of 125 kd and contained an active tyrosine kinase domain. |
| F000008 | AKAP9 | BRAF | 23093608 | This situation also happens for the ANKHD1–PCDH1 fusion in the SK-BR-3 sample. |
| F000009 | ALK | MSN | 22101766 | To further characterize the effects of the ARID1A-MAST2 fusion in MDA-MB-468 cells, we used shRNA targeting MAST2, which displayed efficient knockdown of ARID1A-MAST2 fusion transcript and protein (Fig. S3k–l). |
| F000010 | ANKHD1 | PCDH1 | 17543078 | In addition to wild-type TFE3, ASPSCR1-TFE3 fusion transcripts (three type 1 and two type 2 transcripts) were detected in all cases. |
| F000011 | ARID1A | MAST2 | 12378525 | The BCAS4-BCAS3 fusion transcript was detected only in MCF7 cells, but the BCAS4 gene was also overexpressed in nine of 13 breast cancer cell lines. |
| F000012 | ASPSCR1 | TFE3 | 27095369 | Monitoring BCR-ABL1 fusion transcripts has been widely used to reflect the molecular response to ABL inhibitor therapies and the progression of the disease for patients. |
| F000013 | ATF1 | EWSR1 | 27134074 | We report a case of myeloproliferative neoplasm, unclassifiable (MPN-U) with BCR-JAK2 fusion confirmed by molecular studies. |
| F000014 | ATIC | ALK | 20951315 | These structural changes led to the formation of fusion genes RET-PTC, TRK(-T), and BRAF-AKAP9, which originate as a result of intrachromosomal or interchromosomal rearrangements and are found in papillary thyroid carcinoma. |
| F000015 | BCAS4 | BCAS3 | 21247443 | Eight out of 27 fusion genes (BSG-NFIX, CCDC85C-SETD3, DHX35-ITCH, CMTM7-GLB1, LAMP1-MCF2L, NOTCH1-NUP214, PPP1R12A-SEPT10 and SUMF1-LRRFIP2) identified here were not associated with high-level gene amplifications |
| F000016 | ABL1 | BCR | 9747873 | Both Bcr-Abl fusion proteins exhibit an increased tyrosine kinase activity and their oncogenic potential has been demonstrated using in vitro cell culture systems as well as in in vivo mouse models |
| F000017 | BCR | JAK2 | 18451133 | Although wild-type CANT1 has two alternative first exons (exons 1 and 1a), only exon 1a was detected in CANT1-ETV4 fusion transcripts. A KLK2-ETV4 fusion protein containing the NH2-terminal KLK2 signal peptide would be secreted and could not function as a transcription factor |
| F000018 | BIRC3 | MALT1 | 13679433 | The breakpoint is identical to the one previously reported in the CARS-ALK fusion |
| F000019 | BRD3 | NUTM1 | 23877438 | Although the expression of SLC34A2-ROS1, EZR-ROS1, or KIF5B-RET fusion transcripts was not detected in any of the cases, the expression of CD74-ROS1 fusion transcripts was detected in one (0.9%) of the 114 NSCLCs. |
| F000020 | BRD4 | NUTM1 | 23578175 | Expression of the CCDC6-RET fusion gene in LC-2/ad cells was demonstrated by the mRNA and protein levels, and the genomic break-point was confirmed by genomic DNA sequencing. |
| F000021 | BSG | NFIX | 15026324 | CDH11-USP6 fusion transcripts were demonstrated only in ABC with t(16;17) but other ABCs had CDH11 or USP6 rearrangements resulting from alternate cytogenetic mechanisms. |
| F000022 | C2orf44 | ALK | 11930009 | The in-frame CDK6-MLL transcript is provocative with respect to a potential contribution of the predicted Cdk6-MLL fusion protein in the genesis of the ALL, which also contains an in-frame MLL-AF4 transcript. |
| F000023 | CANT1 | ETV4 | 21813156 | Recent studies have identified a subgroup of undifferentiated soft tissue sarcomas with primitive round to plump spindle cell morphology and a t(4;19)(q35;q13.1) translocation resulting in the expression of a CIC-DUX4 fusion transcript, including 2 tumors previously reported by our laboratory (Cancer Genet Cytogenet 2009;195:1). |
| F000024 | ALK | CARS | 24142740 | The peripheral blood leukocytes revealed the t(2;17;8)(p23;q23;p23) translocation and a CLTC-ALK fusion gene, which have never been reported in BPDCN or in any myeloid malignancies thus far. |
| F000025 | CCDC6 | RET | 12917640 | We report the cloning of a novel clathrin heavy-chain gene (CLTC)-TFE3 gene fusion resulting from a t(X;17)(p11.2;q23) in a renal carcinoma arising in a 14-year-old boy. |
| F000026 | CCND1 | TACSTD2 | 17950782 | COL1A1-PDGFB fusion transcripts have been demonstrated in DFSP and giant cell fibroblastoma as well as their hybrid lesions [4] and [5]. |
| F000027 | CD74 | ROS1 | 15735689 | The first 103 bp of COL1A1 coding sequence are included in the COL1A1-USP6 fusion transcript, but this COL1A1-encoded protein is a short fragment due to a stop codon in the COL1A1 reading frame at the beginning of the USP6-contributed sequence. The novel fusion partners appear well suited to drive USP6 transcription in Proteoglycans; RNA-Binding Proteins; THRAP3 protein, human |
| F000028 | CDH11 | USP6 | 16931951 | Recent cytogenetic and molecular analyses have shown that most LGFMSs have a characteristic chromosomal abnormality, t(7;16)(q33;p11), resulting in the FUS-CREB3L2 fusion gene. |
| F000029 | CDK6 | MLL | 20075182 | Although the CREB3L2-PPARG fusion is rare, its existence points to a limitation of the PPFP RT-PCR assay if the goal is to detect PPARγ fusions as markers of potential thyroid malignancy. |
| F000030 | CHCHD7 | PLAG1 | 17437281 | We analyzed 55 primary salivary gland tumors including 22 mucoepidermoid carcinomas (MECs) to determine the association of MECT1/TORC1/CRTC1-MAML2 fusion transcript to tumor types, level of MEC differentiation and clinicopathologic parameters. |
| F000031 | CIC | DUX4 | 19749740 | We developed reverse transcription-polymerase chain reaction (RT-PCR) assays for CRTC1-MAML2, CRTC2-MAML2, and CRTC3-MAML2 fusions. |
| F000032 | BRAF | CLCN6 | 11894114 | In addition, one of the tumors expressed a cryptic CTNNB1-PLAG1 fusion transcript. |
| F000033 | ALK | CLTC | 15744350 | We found that the t(1;19) in TS-2 fuses the 19p13 gene DAZAP1 (Deleted in Azoospermia-Associated Protein 1) to the 1q23 gene MEF2D (Myocyte Enhancer Factor 2D), leading to expression of reciprocal in-frame DAZAP1/MEF2D and MEF2D/DAZAP1 transcripts. |
| F000034 | CLTC | TFE3 | 18850010 | The FUS-DDIT3 fusion oncogene results from a t(12;16)(q13;p11) chromosome translocation and has a causative role in the initiation of myxoid/round cell liposarcomas (MLS/RCLS). |
| F000035 | CNBP | USP6 | 10222653 | By reverse transcription polymerase chain reaction (RT-PCR) of the RNA from the leukemic cells of the patient, DDX10-NUP98 and NUP98-DDX10 fusion transcripts were detected. |
| F000036 | COL1A1 | PDGFB | 21835007 | Both programs missed two fusion transcripts: DHX35-ITCH and NFS1-PREX1. BT474 RAB22A-MYO9B 20-19 56886176 17256205 8 20 |
| F000037 | COL1A1 | USP6 | 24847761 | In this study, we examined 75 primary CRCs and 121 primary lung cancers in the Japanese population for EIF3E-RSPO2 and PTPRK-RSPO3 fusion transcripts using RT-PCR and subsequent sequencing analyses. |
| F000038 | COL1A2 | PLAG1 | 12642866 | Here we report the first experimental model of MLL. Murine bone marrow cells were retrovirally transduced to express the MLL-eleven nineteen leukemia (MLL-ENL) fusion protein. |
| F000039 | CREB3L2 | FUS | 24285434 | As similar gene fusions were reported in endometrial stromal sarcomas, we screened for potential gene abnormalities in JAZF1 and EPC1 by FISH and found two additional cases with EPC1-PHF1 fusions. |
| F000040 | CREB3L2 | PPARG | 20526349 | Here we used paired-end transcriptome sequencing to screen ETS rearrangement-negative prostate cancers for targetable gene fusions and identified the SLC45A3-BRAF (solute carrier family 45, member 3-v-raf murine sarcoma viral oncogene homolog B1) and ESRP1-RAF1 (epithelial splicing regulatory protein-1-v-raf-1 murine leukemia viral oncogene homolog-1) gene fusions. |
| F000041 | CREBBP | KAT6A | 21193423 | The ETV6-ABL1 fusion gene has also been identified in 3 patients with chronic myeloproliferative neoplasms other than “chronic myeloid leukemia” (cMPN) as well as 7 patients with BCR-ABL1 negative acute lymphoblastic leukemia and 4 patients with acute myeloid leukemia |
| F000042 | CRTC1 | MAML2 | 16572202 | Overproduction of IL3 has been reported in atypical CML following rearrangements of the IL3 gene upstream region in cells from patients with t(5;12) (q23–31;p13) translocation and ETV6-ACSL6 fusion |
| F000043 | CRTC3 | MAML2 | 20033038 | Schematic diagram of the protein domains fused in the predicted ETV6–ITPR2 fusion protein. |
| F000044 | CTNNB1 | PLAG1 | 18068631 | To better understand the cellular origin of breast cancer, we developed a mouse model that recapitulates expression of the ETV6-NTRK3 (EN) fusion oncoprotein, the product of the t(12;15)(p13;q25) translocation characteristic of human secretory breast carcinoma. |
| F000045 | DAZAP1 | MEF2D | 17724745 | To address this, we investigated the presence of FUS-ATF1, EWSR1-ATF1, and the highly related EWSR1-CREB1 fusion in a group of nine AFHs. |
| F000046 | DDIT3 | FUS | 22570737 | The first is a genetic action of the EWSR1-DDIT3 fusion protein, which results in binding to the functional C/EBP site within Opn and Col11a2 promoters through interaction of its DNA-binding domain and subsequent interference with endogenous C/EBPβ function. |
| F000047 | DDX10 | NUP98 | 23329308 | Many studies showed that EWSR1/FEV, EWSR1/FLI and EWSR1/ERG fusion proteins played similar roles. EWSR1/SP3 t(2;22)(q31;q12) SP3 is a transcription factor belonging to the Sp/XKLF family able to recognize GCrich DNA motifs, found in many promoters and enhancers of housekeeping genes |
| F000048 | DDX5 | ETV4 | 9858836 | The discovery of this translocation suggested that there might be a novel EWSR1-ETV4 fusion gene. |
| F000049 | EIF3E | RSPO2 | 23480895 | Fluorescence in situ hybridization mapping suggested the involvement of each of the 2 partner genes, and reverse transcriptase polymerase chain reaction revealed an in-frame EWSR1-NFATC1 transcript. |
| F000050 | ELL | MLL | 22467249 | In addition, rare cases of Ewing sarcoma where EWSR1 becomes fused to another type of transcription factor have been reported: inv(22)(q12q12) resulted in an EWSR1-PATZ1 (POZ/BTB and A-T-hook containing zinc finger 1) fusion gene |
| F000051 | ALK | EML4 | 18383210 | A novel EWSR1-PBX1 fusion gene consisting of exons 1-8 of the 5'-end of EWSR1 and exons 5-9 of the 3'-end of PBX1 was shown to result from the translocation. |
| F000052 | EPC1 | PHF1 | 20815032 | A EWSR1-POU5F1 fusion was identified in a pediatric soft tissue tumor by 3'Rapid Amplification of cDNA Euds (RACE) and subsequently confirmed in four additional soft tissue tumors in children and young adults. |
| F000053 | ERC1 | RET | 21113140 | Mapping analysis demonstrated that deletion of the C-terminus (SLIDE or SANT motives) of hSNF2H impaired, and deletion of the SNF2_N domain fully abrogated NIH3T3 cell transformation by EWSR1-SMARCA5. |
| F000054 | ESRP1 | RAF1 | 24388397 | Common genomic events (i.e., trisomy 3 and extra EWSR1-WT1 and WT1-EWSR1 copies) probably contributed to disease pathogenesis and/or evolution of DSRCT. |
| F000055 | ABL1 | ETV6 | 19760602 | How the new fusion gene contributes to tumorigenesis is unknown, but the finding of an EWSR1 rearrangement suggests that this, possibly even the EWSR1-ZNF444, is a defining pathogenetic feature of at least a subset of these tumors |
| F000056 | ETV6 | ITPR2 | 21424530 | Functional characterization of the novel FAM131B-BRAF fusion demonstrated constitutive MEK phosphorylation potential and transforming activity in vitro. |
| F000057 | ETV6 | JAK2 | 24345920 | Additionally, a FCHSD1-BRAF fusion was identified in a large congenital melanocytic nevus (LCMN) (13). |
| F000058 | ETV6 | NTRK3 | 11739186 | The study demonstrates that the BCR-FGFR1 fusion may occur in patients with apparently typical CML. |
| F000059 | ETV6 | RUNX1 | 22837387 | To determine whether other intrachromosomal FGFR-TACC fusion combinations exist in human GBM, we screened cDNA from an independent panel of 88 primary GBMs and discovered two additional cases (one harboring FGFR1-TACC1 and one FGFR3-TACC3), corresponding to 3 of 97 total GBMs (3.1%), including the GBM-1123 case |
| F000060 | CREB1 | EWSR1 | 21394649 | The TCEA1-PLAG1, HMGA2-FHIT, and HMGA2-NFIB fusion transcripts were not detected. |
| F000061 | DDIT3 | EWSR1 | 16502585 | We recently identified the FIP1L1-PDGFRA fusion gene in approximately 50% of HES/CEL cases. |
| F000062 | ERG | EWSR1 | 22570254 | Genetic characterization of these ALK-positive tumors indicated that full-length ALK expression in two serous carcinoma patients is consistent with ALK gene copy number gain, whereas a stromal sarcoma patient carries a novel transmembrane ALK fusion gene: FN1-ALK. |
| F000063 | ETV1 | EWSR1 | 25806826 | PAX3-FOXO1 (PAX3-FKHR) is the fusion protein produced by the genomic translocation that characterizes the alveolar subtype of Rhabdomyosarcoma, a pediatric sarcoma with myogenic phenotype. |
| F000064 | EWSR1 | FEV | 18094413 | Either FUS-ATF1 or EWSR1-ATF1 have been detected in the few cases published, pointing to the interchangeable role of FUS and EWSR1 in this entity. |
| F000065 | EWSR1 | FLI1 | 15640831 | Cytogenetic analyses have identified a recurrent balanced translocation t(7;16) (q32-34;p11), later shown by molecular genetic approaches to result in a FUS/CREB3L2 fusion gene |
| F000066 | EWSR1 | NFATC2 | 16651630 | Most MLS/RCLS carry a t(12;16) translocation, resulting in a FUS-DDIT3 fusion gene. |
| F000067 | EWSR1 | NR4A3 | 26148230 | In acute myeloid leukemias harboring t(16;21), ERG function is deregulated due to a fusion with FUS/TLS resulting in the expression of a FUS-ERG oncofusion protein |
| F000068 | EWSR1 | PATZ1 | 23052255 | We also report in lung cancer the GOPC-ROS1 fusion originally discovered and characterized in a glioma cell line. |
| F000069 | EWSR1 | PBX1 | 15642402 | In this case, we show that, even with seemingly normal chromosome 8 on conventional cytogenetic analysis, the joining of 8q12.1 to 8q24.1, with subsequent PLAG1-HAS2 fusion, occurred. |
| F000070 | EWSR1 | POU5F1 | 24839999 | Recently, two fusion genes were described in mesenchymal chondrosarcomas: a recurrent HEY1-NCOA2 found in tumors that had not been cytogenetically characterized and an IRF2BP2-CDX1 found in a tumor carrying a t(1;5)(q42;q32) translocation as the sole chromosomal abnormality. |
| F000071 | EWSR1 | SMARCA5 | 19136943 | A fusion between exon 2 of EIF4E2 with exon 8 of HJURP generated the fusion transcript EIF4E2-HJURP and a fusion between exon 9 of HJURP with exon 25 of INPP4A yielded HJURP-INPP4A. Additionally, we found exon 10 of RC3H2 fused to exon 20 of RGS3. One chimeric transcript from Met 3 involves exon 9 of STRN4 with exon 2. Exon 1 of USP10 (red) is fused with exon 3 of ZDHHC7 (green |
| F000072 | EWSR1 | SP3 | 26980027 | We hereby report an unusual gastric tumor arising from the pyloric wall of the stomach in a 9-year old child harboring the exceptionally rare translocation t(7;12) resulting in ACTB-GLI1 gene fusion |
| F000073 | EWSR1 | WT1 | 19528502 | Although the expression of HMGA2-LPP fusion gene has been reported in lipomas, the reciprocal LPP-HMGA2 fusion gene has rarely been described. |
| F000074 | EWSR1 | YY1 | 19837271 | We describe here the fourth reported case of lipoma showing a HMGA2-NFIB fusion, and the first one in a child. |
| F000075 | EWSR1 | ZNF384 | 17171686 | However, in the pleomorphic adenoma expressing the HMGA2/WIF1 fusion transcript, we observed re-expression of HMGA2 wild-type transcripts and very low levels of WIF1 expression. |
| F000076 | BRAF | FAM131B | 23185413 | In our hospital's archives three more cases of MC were found, and we examined them looking for the supposedly more common HEY1-NCOA2 fusion, finding it in all three tumours but not in the case showing t(1;5) and IRF2BP2-CDX1 gene fusion |
| F000077 | BCR | FGFR1 | 16034466 | A PCM1-JAK2 fusion was recently characterised in MPDs |
| F000078 | FGFR1 | PLAG1 | 18722875 | A JAZF1/PHF1 fusion gene was recently found in two tumors showing an exchange between 6p and 7p rearrangement. |
| F000079 | FGFR1 | TACC1 | 26879382 | To date the JAZF1/SUZ12 gene fusion is by far the most frequent and seems to be the cytogenetic hallmark of ESN and LG-ESS. |
| F000080 | FGFR3 | TACC3 | 21884820 | KIAA1549-BRAF fusion transcripts have been detected in frozen tissue, however, methods for FFPE tissue have not been reported. |
| F000081 | FIP1L1 | PDGFRA | 22327624 | The KIF5B-RET fusion leads to aberrant activation of RET kinase and is considered to be a new driver mutation of LADC because it segregates from mutations or fusions in EGFR, KRAS, HER2 and ALK, and a RET tyrosine kinase inhibitor, vandetanib, suppresses the fusion-induced anchorage-independent growth activity of NIH3T3 cells. |
| F000082 | ALK | FN1 | 22347464 | With this system, we successfully identified a novel ALK fusion, KLC1-ALK. |
| F000083 | FOXO1 | PAX3 | 21036922 | To our knowledge, this is the first description of a KLK2–ETV1 fusion event. |
| F000084 | FRYL | MLL | 19144982 | One patient had an MLL-ACTN4 fusion, 2 others an MLL-TET1 fusion |
| F000085 | ATF1 | FUS | 10850414 | The presence of coiled-coil domains in the resulting ktn1/ret fusion protein suggests ligand-independent dimerization and thus constitutive activation of the ret TK domain. |
| F000086 | CREB3L1 | FUS | 23630011 | A LIFR-PLAG1 fusion was detected by RACE and then confirmed by FISH in one soft tissue ME tumor with tubular formation. |
| F000087 | ERG | FUS | 26888508 | MALAT1-TFEB fusion gene was identified in 2 cases by polymerase chain reaction and direct sequencing. |
| F000088 | GOPC | ROS1 | 12461747 | To focus on the identification of MLL-ACTN4 as a rare but recurrent MLL rearrangement, we present 2 cases of MLL-ACTN4 rearrangement, and compare these patients with regard to diagnostic findings and clinical courses. |
| F000089 | HAS2 | PLAG1 | 18617060 | Sequence analysis of reverse-transcriptional polymerase chain reaction product revealed a novel variant form of MLL-ELL transcript in which MLL exon 10 was fused to ELL exon 3. |
| F000090 | HEY1 | NCOA2 | 20980053 | To check the expression of the MLL-EP300 fusion transcript in the bone marrow cells, reverse transcription PCR (RT-PCR) was performed |
| F000091 | HMGA2 | LPP | 23091311 | Fifteen fusion transcripts were included: BCR-ABL1, PML-RARA, ZBTB16-RARA, RUNX1-RUNX1T1, CBFB-MYH11, DEK-NUP214, TCF3-PBX1, ETV6-RUNX1, MLL-AFF1, MLL-MLLT4, MLL-MLLT3, MLL-MLLT10, MLL-ELL, MLL-MLLT1, and MLL-MLLT6 |
| F000092 | HMGA2 | NFIB | 22952941 | The resulting fusion genes, PICALM-AF10 and MLL-PICALM, have been found in aggressive hematologic malignancies |
| F000093 | HMGA2 | WIF1 | 18492691 | Molecular analysis led to the identification of several MLL-SEPT6 fusion transcripts in all cases, including a novel MLL-SEPT6 rearrangement (MLL exon 6 fused with SEPT6 exon 2) |
| F000094 | HOOK3 | RET | 24323992 | In the April 2013 issue of Haematologica, Lee et al. have described the TET1 genomic breakpoints and clinical features of MLL-TET1 rearranged cases of acute leukemia |
| F000095 | CDX1 | IRF2BP2 | 23150706 | Of the 13 patients, nine patients had KIF5B-RET, three patients had CCDC6-RET, and one patient had a novel NCOA4-RET fusion |
| F000096 | JAK2 | PAX5 | 19318479 | To detect the NFATc2-EWSR1 fusion transcript, the primers NFATc2_867_F and EWSR1_1561_R were used. |
| F000097 | JAZF1 | PHF1 | 24140425 | Initially, three independent cases of MAST gene fusions were identified by transcriptome analyses-ARID1A-MAST2, ZNF700-MAST1, and NFIX-MAST1 |
| F000098 | JAZF1 | SUZ12 | 15087377 | RT-PCR reactions to check the putative NIN-PDGFRB translocation were carried out with Immolase DNA polymerase heat activated (Bioline; London, United Kingdom) using NIN-1 to NIN-6 as forward primers and PDGFRB-1 and PDGFRB-2 as reverse primers in separate and multiple combinations. |
| F000099 | KIF5B | RET | 24962792 | Finally, using quantitative reverse transcriptase PCR and immunohistochemistry (IHC) we identified the TPM3-NTRK1 rearrangement in a CRC clinical sample, therefore suggesting that this chromosomal translocation is indeed a low frequency recurring event in CRC and that such patients might benefit from therapy with TRKA kinase inhibitors |
| F000100 | ALK | KLC1 | 10074915 | A single tumor exhibited a TPR/NTRK1 fusion (TRK-T2) |
