# Supplementary material for: ProtFus: A Comprehensive Method Characterizing Protein-Protein Interactions of Fusion Proteins
Source: PLoS Comput Biol. 2019 Aug 22;15(8):e1007239. doi: 10.1371/journal.pcbi.1007239 (PMC6705771; doi:10.1371/journal.pcbi.1007239)
Supplement: S6 Table — (DOCX) [file pcbi.1007239.s006.docx]

**Supplementary Table S6**

**ProtFus: A Comprehensive Method for Characterizing Protein-Protein Interactions of Fusion Proteins**

Somnath Tagore^1,3^, Alessandro Gorohovski^1^, Lars Juhl Jensen^2^ and Milana Frenkel-Morgenstern^1,*^

^1^ The Azrieli Faculty of Medicine, Bar-Ilan University, 8 Henrietta Szold St, Safed 13195, ISRAEL

^2^ Cellular Network Biology Group, The Novo Nordisk Foundation Center for Protein Research, University of Copenhagen, DENMARK

^3^ Present Address: Department of Systems Biology, Columbia University, New York, NY, 10032, USA.

*Corresponding Author E-mail: [milana.morgenstern@biu.ac.il](mailto:milana.morgenstern@biu.ac.il)

**Table S6: Fusion PPIs tokens identified by ProtFus for 100 PubMed IDs**

| FP_ID | PubMed | Interaction Description |
| --- | --- | --- |
| F000001 | 23719267 | In mice, the FIG-ROS1 fusion gene has been shown to promote the formation of astrocytomas when ectopically expressed in the basal ganglia, and the EZR-ROS1 fusion gene has been shown to promote lung adenocarcinoma when ectopically expressed in lung epithelium |
| F000002 | 18594527 | Expression of ACSL3 was also elevated in a panel of ‘androgen-sensitive' (LAPC-4, LNCaP, MDA PCa2a, MDA PCa2b, and 22Rv1) versus ‘androgen-insensitive' (PPC1, PC3, and DU145) prostate cancer cell line |
| F000003 | 12138125 | We found that co-expression of Dyrk1 and Gli1 strongly induced Gli1-dependent gene transcription in the presence of the 3′GliBS-Luc reporter construct, but not in the presence of the mutant reporter construct, m3′GliBS-Luc |
| F000004 | 26288717 | Recently, Ehrlicher and Pollak et al. demonstrated that in FSGS, a K255E mutation in ACTN4 changes the cellular biological properties in which increasing the affinity for actin increases cellular forces and work and decreases cellular movement |
| F000005 | 11886378 | Hybridization of the MLL/AF4 probe combination to pure SEM cells resulted in 87% of the cells displaying the expected AF4x3/MLLx3/AF4 con MLLx2 hybridization pattern |
| F000006 | 23762276 | Several genes involved in fusions have been reported to be fused or rearranged in other cases–AGPAT5, NOTCH2, PUM1, SEC22B, SGK1 and TRERF1 (all early or unclassified), while several are mutated at sequence level, notably SYNE2 |
| F000007 | 20613748 | The finding of mutated RAF genes in prostate cancer is consistent with a previous observation that oncogenic BRAFV600E can initiate prostate cancer in mouse models16 and may have major implications for therapy |
| F000008 | 23543667 | The large amplification on chromosomes 7 and 12 could potentially activate the RAS-RAF-MEK pathway by amplification of the BRAF gene located on chromosome 7 or amplification of KRAS located on chromosome 12 |
| F000009 | 11310834 | These findings indicate that MSN may act as an alternative fusion partner for activation of ALK in ALCL and provide further evidence that oncogenic activation of ALK may occur at different intracellular locations |
| F000010 | 23093608 | BWA found six discordant alignments with MAPQ = 0 between ANKHD1 and both PCDH1 and ANKHD1–EIF4EBP3 (ENSG00000254996). |
| F000011 | 21622959 | Juxtaposition of the ARID1A promoter would place control of MAST2 which is downstream of the RB1 pathway, as evidenced by the preponderance of E2F sites in the ARID1A promoter and by the observation that ARID1A is regulated in a cell cycle-dependent manner |
| F000012 | 23828314 | In response, we demonstrate through both validation and extensive clinical experience that a break-apart strategy probe set for TFE3, including a chromosome X centromere probe as a control and a TFE3/ASPSCR1 dual-color, single-fusion reflex probe set, is an excellent test to aid in the identification of tumors with Xp11.2 rearrangement. |
| F000013 | 15884099 | Simultaneous expression of the EWSR1-ATF1 and MITF-M transcripts in CCS has led to the proposal that the MITF-M promoter is transactivated by EWSR1-ATF1 |
| F000014 | 20554525 | Grb2 has been shown to bind NPM-ALK and ATIC-ALK in previous works |
| F000015 | 21305644 | In the remaining cell lines, two fusion genes, BCAS4-BCAS3 and CCDC6-RET, were detected from the breast and thyroid cancer cell lines, MCF-7 and TPC-1, respectively, and thus validating the microarrays ability to detect fusion genes outside the group of positive controls |
| F000016 | 9747873 | The SH2-containing adapter protein GRB10 interacts with BCR-ABL |
| F000017 | 23904814 | It was demonstrated by preclinical studies that BCR-JAK2 induces STAT5 activation and elicits BCRxL gene expression |
| F000018 | 20440281 | The BIRC3–MALT1 fusion protein and the overexpressed MALT1 protein in the t(14;18) have been shown to activate NF-κB and thereby promote cellular proliferation and resistance to apoptosis |
| F000019 | 18406326 | Brd2- and Brd3-associated chromatin is significantly enriched in H4K5, H4K12, and H3K14 acetylation and contains relatively little dimethylated H3K9 |
| F000020 | 26551281 | In contrast, BRD4 is an important member of the bromodomain and extra-terminal domain proteins (the BET family) known to regulate cell cycle progression, survival signaling, chromatin structure, epigenetic memory and embryonic stem cell development |
| F000021 | 21247443 | Interphase FISH showing amplified signals of BSG and NFIX (left) and NOTCH1 and NUP214 (right) in KPL-4 |
| F000022 | 22327622 | Complementary DNA (cDNA) sequencing identified 75 read pairs spanning the fusion junction (data not shown) and an 89.8-fold increase in 3′ ALK expression beginning at exon 20 relative to exons 1–19, suggesting that the C2orf44-ALK fusion transcript results in ALK kinase overexpression |
| F000023 | 18451133 | Exclusive usage of CANT1 exon 1a as first exon in CANT1-ETV4 fusion transcripts might have various explanations, including the positions of breakpoints of the specific genomic rearrangement and the prostate-specific expression of transcripts starting at exon 1a |
| F000024 | 13679433 | This alternative splicing occurs downstream of the breakpoint identified in IMT and does not affect the sequence of the CARS-ALK chimeric protein |
| F000025 | 23150705 | The fusion of the intracellular kinase-encoding domain of RET to CCDC6 and NCOA4, among others, gives rise to ligand-independent activation of RET. |
| F000026 | 23382248 | Expression of an RNA chimera fusing CCND1 and TROP2 (TACSTD2) transcripts has been demonstrated to result in immortalization and transformation of human epithelial cells |
| F000027 | 22215748 | To confirm this activity, we showed that crizotinib also inhibits ROS1 phosphorylation in HEK 293 cells transfected with a CD74-ROS1 fusion gene expression construct |
| F000028 | 23769422 | Interestingly, we have not encountered the fusion CDH11-USP6 in NF or soft tissue ABC, and the only soft tissue ABC characterized at the cytogenetics level showed a chromosomal rearrangement consistent with the presence of COL1A1-USP6 |
| F000029 | 11930009 | The central role of Cdk6 in cell cycle progression and its recurrent alteration in human cancer suggest that the CDK6-MLL juxtaposition may have been a cooperating mutation in leukemogenesis in patient 38 |
| F000030 | 16736500 | The fact that both the CHCHD7-PLAG1 and TCEA1-PLAG1 fusions are caused by cytogenetically cryptic rearrangements in tumors with different karyotypic abnormalities or normal karyotypes indicates that PLAG1 gene fusions are more common than originally suggested by conventional cytogenetics |
| F000031 | 21813156 | One CIC-DUX4–positive tumor showed membranous CD99 positivity, 2 showed focal S100 positivity, and 1 showed focal CD57 positivity |
| F000032 | 23817572 | Another new BRAF alteration was identified in ICGC_PA65, resulting in a three amino acid insertion (p.R506_insVLR) in the interdomain cleft of BRAF - a structural region linked to its activity17 and homodimerization |
| F000033 | 24142740 | The tyrosine kinase domain of ALK is constitutively phosphorylated by the formation of CLTC-ALK, the tumorigenicity of which has been verified in vitro and in vivo |
| F000034 | 12917640 | CLTC encodes a major subunit of clathrin, a multimeric protein on cytoplasmic organelles, and is a known recurrent fusion partner of the ALK tyrosine kinase gene in anaplastic large-cell lymphoma and inflammatory myofibroblastic tumors |
| F000035 | 15735689 | The novel fusion partners appear well suited to drive USP6 transcription in the bone/mesenchymal context: osteomodulin is expressed strongly in osteoblastic lineages, and the COL1A1 promoter has an oncogenic role in the mesenchymal cancer dermatofibrosarcoma protuberans |
| F000036 | 11420709 | In addition, COL1A1-PDGFB transfected cell supernatants significantly stimulated fibroblastic cell growth, through the activation of the PDGFB receptor pathway |
| F000037 | 15735689 | Since the point of fusion is highly specific for PDGFB but spread over almost the entire locus for COLlAl, the role of the COL1A1 gene may be simply to up-regulate the expression of PDGFR, which acts as an auto- or paracrine growth factor |
| F000038 | 10987300 | Consequently, COL1A2-PLAG1 encodes a full-length PLAG1 protein and a short, COOH-terminal-truncated, COL1A2 protein |
| F000039 | 21092583 | Thus, it is expected that the novel FUS/CREB3L1 chimera will have a similar impact at the cellular level as the much more common FUS/CREB3L2 fusion protein. |
| F000040 | 20075182 | Although the CREB3L2-PPARG fusion is rare, its existence points to a limitation of the PPFP RT-PCR assay if the goal is to detect PPARγ fusions as markers of potential thyroid malignancy |
| F000041 | 24798186 | Although no samples in our series were positive for MLL-ELL, MLL-MLLT1, ZBTB16-RARA, RBM15-MKL1, or KAT6A-CREBBP, probably because of the low frequency of occurrence of these fusion genes, 6, 7, 9, 24, 25 and 26 the ability of this method to detect these rearrangements was verified by testing positive controls for each of them |
| F000042 | 17334997 | The CRTC1-MAML2 fusion protein acts by inducing transcription of cAMP/CREB target genes, and this activity is crucial for the transforming properties of the protein. |
| F000043 | 18050304 | Both gene fusions seem to result in an identical tumor phenotype and the fusion genes CRTC1-MAML2 and CRTC3-MAML2 may play a similar role in the development of mucoepidermoid carcinomas. |
| F000044 | 10029085 | The t(3;8) results in promoter swapping between PLAG1 and the constitutively expressed gene for beta-catenin (CTNNB1), leading to activation of PLAG1 expression and reduced expression of CTNNB1. |
| F000045 | 15182431 | In addition, exogenous expression of MEF2D-DAZAP1 and DAZAP1-MEF2D promoted the growth of HeLa cells. |
| F000046 | 20017906 | In addition, CDK2 showed an increased affinity for cytoskeletal proteins in cells expressing FUS-DDIT3 and DDIT3. |
| F000047 | 23224603 | Although two reciprocal chimeric products, NUP98-DDX10 and DDX10-NUP98, were predicted, only NUP98-DDX10 appears to be implicated in tumorigenesis. |
| F000048 | 18794152 | Therefore, the FLJ35294-ETV1 and CANT1-ETV4 fusions can be categorized as class II gene fusions in prostate cancer which include rearrangements involving fusions from prostate-specific androgen-induced 5′ partner genes (21); whereas DDX5-ETV4 may represent a class IV gene fusion, in which non-tissue-specific promoter elements drive ETS gene expression |
| F000049 | 24847761 | Although the expression of EIF3E-RSPO2 or PTPRK-RSPO3 was not detected in any of the NSCLCs, EIF3E-RSPO2 and PTPRK-RSPO3 fusion transcripts were detected in two CRCs and one CRC, respectively |
| F000050 | 10995463 | These studies demonstrate the enhancing effect of MLL-ELL on the proliferative potential of myeloid progenitors as well as its causal role in the genesis of acute myeloid leukemias. |
| F000051 | 18594010 | TAE684 inhibited the growth of one of three (H3122) EML4-ALK-containing cell lines in vitro and in vivo, inhibited Akt phosphorylation, and caused apoptosis. |
| F000052 | 16397222 | The EPC1/PHF1 chimeric fusion led to an open reading frame containing 581 amino acid residues from EPC1, six additional amino acid residues upstream from the initial methionine, and the entire PHF1 protein sequences consisting of 567 amino acids, in total, 1,154 amino acids in the predicted chimeric protein |
| F000053 | 17690697 | Interestingly, ERC1, H4(D10S170) and TPM3 are three PDGFRB partners in myeloid malignancies that are also involved in human papillary thyroid carcinoma: ERC1 and H4(D10S170) fuse with RET as a result of t(10;12)(q21;p13) and of inv(10)(q11.2q21), producing the ERC1-RET and the H4(D10S170)-RET autophosphorylated tyrosine kinase, respectively;4, 5 TPM3 rearranges with the nearby neurotrophic tyrosine kinase receptor type 1 (NTRK1/1q23) gene |
| F000054 | 20526349 | NIH3T3 cells over-expressing SLC45A3-BRAF formed rapidly growing tumors in nude mice (Fig. 3b); however NIH3T3 cells over-expressing ESRP1-RAF1 did not form tumors (data not shown), which may reflect signaling differences between the different fusion products |
| F000055 | 10500813 | Hyperdiploidy > 50 chromosomes and ETV6-CBFA2 fusions have been used to identify low-risk cases, and BCR-ABL and MLL-AF4 to define high-risk leukemias. |
| F000056 | 20033038 | ETV6-ITPR2, an expressed, in frame fusion gene generated by a 15Mb inversion in the primary breast cancer PD3668a |
| F000057 | 17077140 | Expression of TEL-JAK2 in primary human hematopoietic cells drives erythropoietin-independent erythropoiesis and induces myelofibrosis in vivo. |
| F000058 | 14668342 | A highly conserved NTRK3 C-terminal sequence in the ETV6-NTRK3 oncoprotein binds the phosphotyrosine binding domain of insulin receptor substrate-1 [?]: an essential interaction for transformation. |
| F000059 | 20190817 | ETV6/RUNX1 abrogates mitotic checkpoint function and targets its key player MAD2L1 |
| F000060 | 18094413 | CONCLUSION: We identified the presence of either EWSR1-CREB1 or EWSR1-ATF1 in all the cases, strengthening the concept of chromosomal promiscuity between AFH and clear cell sarcoma. |
| F000061 | 22570737 | Overexpressing EWSR1-DDIT3 under the control of a CMV promoter using the pFLAG-CMV4 EWSR1-DDIT3 expression vector significantly repressed Opn and Col11a2 promoter activities by 79% and 78%, respectively; however, overexpressing EWSR1 and DDIT3 did not |
| F000062 | 8162068 | Identical EWS nucleotide sequences found in the EWS/FLI-1 fusion transcripts are fused to portions of ERG encoding an ETS DNA-binding domain resulting in expression of a hybrid EWS/ERG protein. |
| F000063 | 10523827 | When EWS/ETV1 or EWS/FLI1 expressing NIH3T3 cells are injected into SCID mice, tumors form more often and faster than with NIH-3T3 cells with empty vector controls. |
| F000064 | 17172842 | EWS-FLI, EWS-ERG, and EWS-FEV caused NIH3T3 cells to exhibit anchorage independent growth whereas EWS-ETV1 and EWS-ETV4 did not. |
| F000065 | 8516324 | Deletion of either the EWS domain or the FLI1 corresponding to the DNA-binding domain totally abrogated the ability for EWS-FLI1 to transform 3T3 cells. |
| F000066 | 24993903 | The presence of EWSR1-NFATC2 fusion, focal small-round-cell morphology, and CD99 immunopositivity in the reoccurrence favor an Ewing-like sarcoma |
| F000067 | 18855877 | Western blots further show that an isoform of the native NR4A3 receptor lacking the C-terminal domain is very highly expressed in tumours positive for EWSR1/NR4A3, and co-transfections of this isoform along with EWSR1/NR4A3 indicate that it may negatively regulate the activity of the fusion protein on the PPARG promoter |
| F000068 | 22467249 | The EWSR1 gene encodes a multifunctional protein, member of the ten-eleven translocation (TET) family of proteins, that is involved in various cellular processes, including gene expression, cell signalling and ribonucleic acid (RNA) processing and transport |
| F000069 | 18383210 | In the EWSR1-PBX1 fusion gene detected, the 5′ transactivation domain of EWSR1 and the 3′ DNA-binding domain of PBX1 were retained EWSR1-PBX1 |
| F000070 | 20203285 | Knockdown of EWS-POU5F1 in the t(6;22) sarcoma-derived GBS6 cell line resulted in a significant decrease of cell proliferation because of G1 cell cycle arrest associated with p27(Kip1) up-regulation. |
| F000071 | 21113140 | The EWSR1–SMARCA5 chimeric cDNA in the LITMUS38i vector was amplified by PCR to add the epitope tag FLAG and the Kozak consensus translation initiation sequences into the N-terminal region |
| F000072 | 23329308 | SP3 is a transcription factor belonging to the Sp/XKLF family able to recognize GCrich DNA motifs, found in many promoters and enhancers of housekeeping genes |
| F000073 | 12498708 | In this issue of Cancer Cell, it show that the resulting EWS-WT1 gene-fusion product leads to overexpression of BAIAP3, a protein implicated in regulated exocytosis. |
| F000074 | 23630070 | The putative EWSR1–YY1 encoded protein would contain, as other EWSR1-fusion-encoded proteins, the transactivation domain of EWSR1 and the DNA-binding domain of YY1 |
| F000075 | 23329308 | Transcription factor ZNF384/CIZ/NMP4 plays a role in bone metabolism and spermatogenesis |
| F000076 | 25040262 | In vitro experiments demonstrated that FAM131B-BRAF is also an activator of the MAPK pathway |
| F000077 | 11746971 | By analogy with data obtained from previously characterized fusion genes involving FGFR1 and BCR/ABL, it is likely that the oligomerization domain contributed by BCR is critical and that its dimerizing properties lead to aberrant FGFR1 signaling and neoplastic transformation. |
| F000078 | 18059337 | Using primers located in exon 1 of FGFR1 and in exon 4 of PLAG1, we could show that 9 of the 10 cases with r(8) expressed FGFR1–PLAG1 fusion transcripts |
| F000079 | 21305644 | Quantitative analyses of mitoses revealed that cells expressing FGFR3-TACC3 or FGFR1-TACC1 exhibit three to five times more errors in chromosomal segregation compared with control cells |
| F000080 | 26425723 | Comprehensive genomic profiling of the original cervical biopsy was pursued to identify additional therapeutic options and revealed the following: FGFR3–TACC3 fusion (breakpoints at FGFR3 intron 18 and TACC3 intron 7), BRAF 3′ tandem duplication (breakpoint in intron 9 with duplication of exons 10–18), activating PIK3CA missense mutation (E545K), CDNK2A loss, and subclonal activating missense mutations in KRAS (G12C), and HRAS (G13R) |
| F000081 | 12842979 | Recently, a novel tyrosine [?] kinase that is generated from fusion of the Fip1-like 1 (FIP1L1) and PDGFR alpha (PDGFRA) genes has been identified as a therapeutic target for imatinib mesylate in hypereosinophilic syndrome (HES). |
| F000082 | 22570254 | Encoded by the first 23 exons, the FN1 portion of FN1-ALK retains a diverse set of binding domains involved in fibronectin self-association and interaction with other ECM components, which could potentially provide strong activating signal to ALK |
| F000083 | 25806826 | PAX3-FOXO1 may contribute to tumor formation by inhibiting the tumor suppressor activities which are characteristic of both FOXO family members and TGF-β pathways |
| F000084 | 18195096 | MEIS1 expression was substantially greater in all of the leukemia cases than in the cells with MLL-FRYL |
| F000085 | 16849546 | Because most sarcomas bearing unique chromosomal translocations are believed to originate from common progenitor cells, and because MPCs populate most organs, we expressed the sarcoma-associated fusion proteins FUS/TLS-CHOP, EWS-ATF1, and SYT-SSX1 in MPCs and tested the tumorigenic potential of these cells in vivo. |
| F000086 | 15640831 | The case with the variant FUS/CREB3L1 fusion also had a break in exon 5 of the CREB gene, but as the breakpoint in FUS was in exon 9, a larger portion of FUS was included in the fusion gene |
| F000087 | 26148230 | We found that FUS–ERG mainly binds non-promoter regions in a complex consisting of other ETS factors, GATA2, LMO2, LYL1, RUNX1, TAL1 and RNAPII |
| F000088 | 23052255 | The GOPC-ROS1 fusion protein has been shown to have constitutively active kinase activity and its transforming potential has been demonstrated in a mouse transgenic model where it resulted in glioblastomas in an Ink4a;Arf-null background |
| F000089 | 15642402 | In this case, we show that, even with seemingly normal chromosome 8 on conventional cytogenetic analysis, the joining of 8q12.1 to 8q24.1, with subsequent PLAG1-HAS2 fusion, occurred |
| F000090 | 24839999 | The absence of HEY1-NCOA2 fusion in some cases has been explained as being due to methodological inadequacy (16,19) but the possibility of other disease-specific fusion gene(s), and thus pathogenetic heterogeneity in this diagnostic entity, should not be ruled out |
| F000091 | 16375854 | These results indicate that in vivo overexpression of HMGA2-LPP promotes chondrogenesis by upregulating cartilage-specific collagen gene expression through the N-terminal DNA binding domains. |
| F000092 | 19837271 | In the pediatric lipoma studied here, the sequence analysis of the HMGA2–NFIB fusion gene revealed that the chimeric transcript was identical to some described previously in pleomorphic adenomas of the salivary glands [24] and in lipomas |
| F000093 | 19837271 | Taken together these data suggest that in pleomorphic adenoma PA37 cells, the identified HMGA2 fusion transcripts containing intronic HMGA2 sequences are expressed at lower levels than the HMGA2/WIF1 fusion transcript |
| F000094 | 17639057 | The ability of HOOK3-RET protein to induce cell transformation was studied in an NIH3T3 cells focus assay |
| F000095 | 23185413 | The IRF2BP2-CDX1 fusion is thus suggested to take part in MC tumorigenesis and/or progression |
| F000096 | 25515960 | PAX5-JAK2 binds to PAX5 target loci and activates these genes |
| F000097 | 18722875 | In the present study, we show that the low-grade endometrial stromal sarcoma cell line JHU-ESS1, established by Fresia et al. [9], which carries a der(7)t(6;7)(p21;p22), also harbors a JAZF1/PHF1 fusion gene |
| F000098 | 26879382 | One out of six samples was positive both by RT-PCR and by FISH, indicating lower prevalence of JAZF1/SUZ12 gene fusion in extrauterine in comparison to uterine ESSs |
| F000099 | 22327624 | The LADCs that were positive for the KIF5B-RET fusion showed twofold to 30-fold higher RET expression than non-cancerous lung tissues |
| F000100 | 22347464 | Infection of 3T3 cells with the virus expressing KLC1-ALK readily produced multiple transformed foci in culture and subcutaneous tumors in a nude mouse tumorigenicity assay (Figure 4), confirming the potent transforming ability of KLC1-ALK |
